# Development of a picture‐based tool for subjective deficits in cognition and ADLs

**DOI:** 10.1002/dad2.70413

**Published:** 2026-06-29

**Authors:** Isabell Ballasch, Ronja Faßbender, Onur A. Oezguer, Sophia Felicitas Schuh, Nils Richter, Esra Kara, Anna Sauerbier, Ulrike Lambotte, Haidar S. Dafsari, Jutta Stahl, Gereon Rudolph Fink, Elke Kalbe, Josef Kessler, Stefanie Theresa Jost

**Affiliations:** ^1^ Department of Neurology University of Cologne Faculty of Medicine and University Hospital Cologne Cologne Germany; ^2^ Department of Medical Psychology, Neuropsychology & Gender Studies Center for Neuropsychological Diagnostics and Intervention (CeNDI) Faculty of Medicine and University Hospital Cologne University of Cologne Cologne Germany; ^3^ Cognitive Neuroscience Institute of Neuroscience and Medicine (INM‐3), Jülich Research Center Jülich Germany; ^4^ Department of Psychology University of Cologne Cologne Germany

**Keywords:** activities of daily living, ADL, dementia, language abilities, MCI, mild cognitive impairment, NCD, neurocognitive disorder, picture‐based, SCD, self‐report questionnaire, subjective cognitive decline

## Abstract

**INTRODUCTION:**

Traditional instruments assessing activities of daily living (ADLs) and cognitive complaints are language based and insufficiently capture technology‐based ADLs, limiting use among individuals with limited language proficiency or language impairments. We developed the picture‐based assessment of subjective deficits in cognition and ADL (Pic‐ADL) to address this gap and reflect contemporary everyday functioning.

**METHODS:**

The Pic‐ADL is a picture‐based self‐report questionnaire covering cognitive and ADL complaints, including novel items on digital media. We investigated 243 healthy controls and 70 neurological patients, who completed neuropsychological testing and language‐based questionnaires for validation.

**RESULTS:**

The Pic‐ADL showed high acceptability, reliability, and validity. Patients with major neurocognitive disorder (NCD) reported significantly more difficulties than patients with mild NCD and controls (_p_η^2 ^= .49). Classification accuracy of controls/mild NCD versus major NCD was 91% (sensitivity = .83, specificity = .86).

**DISCUSSION:**

The Pic‐ADL demonstrated strong psychometric properties and may be useful in settings where language‐based assessment is challenging, supporting improved NCD diagnosis.

**TRIAL REGISTRATION**: Trial registration number: DRKS00033038 (date of registration: 27.11.2023)

**TRIAL REGISTRY**: German Clinical Trials Register (Deutsches Register Klinischer Studien; DRKS)

## Highlights

- Development of a picture‐based tool for cognitive and activity of daily living (ADL) complaints (**Pic‐ADL**).
- Modern ADL items on smartphone, computer, and internet‐related tasks.
- Pic‐ADL shows high reliability and validity in controls and neurological patients.
- High accuracy in classifying mild versus major neurocognitive disorder.

## 1 BACKGROUND

The diagnostic criteria for mild cognitive impairment (MCI), originally defined by Petersen et al.^1^ and refined by later work,^2^, ^3^, ^4^ emphasize subjective cognitive complaints, objective cognitive impairment, and largely preserved activities of daily living (ADLs).^4^ Conceptually, MCI corresponds to “mild neurocognitive disorder” (mild NCD), as defined in diagnostic frameworks such as the Fifth Edition of the *Diagnostic and Statistical Manual of Mental Disorders* (DSM‐5) and the National Institute on Aging–Alzheimer's Association (NIA‐AA).^5^, ^6^, ^7^ These frameworks distinguish mild from major NCD primarily based on functional independence; whereas major NCD entails loss of independence in ADLs, autonomy is largely preserved in mild NCD,^2^, ^3^ although subtle difficulties may emerge in complex tasks.^8^

Across the spectrum of NCDs, subjectively perceived cognitive and functional difficulties constitute an important component of clinical assessment, providing information on everyday functioning beyond objective testing alone.^9^ Although subjective complaints alone are insufficient to establish a diagnosis,^10^ they nonetheless provide relevant clinical information across different stages of cognitive impairment.^11^ However, existing instruments assessing cognitive complaints and ADLs are language dependent and require adequate reading comprehension and abstract reasoning. Individuals with limited language proficiency, acquired language disorders, or low literacy may therefore be disadvantaged and face an increased risk of misdiagnosis.^12^, ^13^ In addition, about 19% of adults in the United States read at or below basic literacy levels.^14^

Furthermore, many widely used instruments do not adequately reflect the digital demands of everyday life. Although 90% of U.S. individuals own a smartphone and most go online multiple times per day,^15^ traditional ADL measures still focus on conventional activities such as telephone use,^16^ and even modern instrumental ADL (IADL) scales often fail to capture digital activities such as smartphone, internet, or social media use,^17^

Thus, there is a clear need for updated tools with limited linguistic or cognitive abilities. To address this gap, we developed the picture‐based assessment of subjective deficits in cognition and ADLs (Pic‐ADL). This study aimed to evaluate its psychometric properties and diagnostic performance in healthy controls and neurological patients.

## 2 METHODS

### 2.1 Participants and Recruitment

**FIGURE 1:**
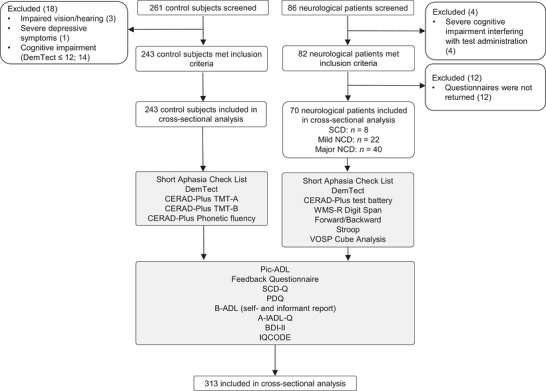
Flow of Participants and Study Procedures. A‐IADL‐Q, Amsterdam Instrumental Activities of Daily Living Questionnaire; B‐ADL, Bayer Activities of Daily Living; CERAD, Consortium to Establish a Registry for Alzheimer's Disease; IQCODE, Informant Questionnaire on Cognitive Decline in the Elderly; NCD, neurocognitive disorder; PDQ, Perceived Deficits Questionnaire; Pic‐ADL, Picture‐based assessment of subjective deficits in cognition and Activities of Daily Living; SCD, subjective cognitive decline; SCD‐Q, Subjective Cognitive Decline Questionnaire; Stroop, Stroop Color‐Word‐Interference Test; TMT‐A, Trail Making Test Part A; TMT‐B, Trail Making Test Part B; VOSP, Visual Object and Space Perception Battery; WMS‐R, Wechsler Memory Scale – Revised.

Data collection for this cross‐sectional study took place between September 2023 and April 2025. In total, *N *= 347 participants (261 controls, 86 neurological patients) were recruited, and the final sample consisted of *N *= 313 participants after exclusions (243 controls, 70 neurological patients). Figure 1 illustrates participant flow and study procedures.

Healthy controls were recruited and assessed by trained medical students through their personal networks in Germany. Inclusion criteria were: (1) age 40–90 years, (2) normal or corrected vision and hearing (self‐report), (3) sufficient German language skills, (4) sufficient language proficiency (≥14 points on the Short Aphasia Check List^18^), (5) no severe depressive symptoms (Beck Depression Inventory‐II [BDI‐II] ≤28^19^), and (6) no neurological disease or cognitive impairment (self‐report). To ensure the absence of clinically relevant cognitive impairment, control participants were excluded if they scored ≤12 on the DemTect, indicating dementia‐range performance.^20^

**RESEARCH IN CONTEXT**

1. **Systematic Review**: We searched the literature using standard scientific databases. Although several instruments assess activities of daily living (ADLs) and subjective cognitive complaints, they are largely language dependent, require intact reading comprehension, and insufficiently reflect modern digital activities. No validated instrument addressing these limitations—particularly reduced linguistic demands and contemporary ADL domains—was identified.
2. **Interpretation**: This study introduces the Pic‐ADL, a predominantly picture‐based screening tool capturing subjective cognitive and ADL impairment, including both traditional ADL items and items on digital media use. Our findings show that the Pic‐ADL is reliable, valid, and able to differentiate individuals with mild and major neurocognitive disorder, thereby extending existing ADL assessments and improving diagnostic applicability in populations with limited language proficiency.
3. **Future Directions**: Future work should evaluate the Pic‐ADL in larger, culturally diverse samples, assess its cross‐linguistic validity, and determine its applicability across clinical subgroups. Development of brief and informant‐based versions should also be pursued to optimize clinical utility.

Neurological patients with subjective or objective cognitive impairment were examined by the staff of the Neuropsychology working group at the Department of Neurology, University Hospital of Cologne, Germany. Except for one inpatient, all patients were assessed as part of the outpatient memory consultation service. Inclusion criteria were: (1) age 40–90 years, (2) normal or corrected vision and hearing (self‐report), (3) sufficient German language skills, and (4) sufficient language proficiency (≥14 points in the Short Aphasia Check List^18^).

Two additional control samples were recruited for validation using the same recruitment criteria as the main sample: *n *= 120 control participants to assess test–retest reliability; *n *= 40 control participants, equally split between versions with and without captions, to examine the impact of picture captions. Additional information on the two control samples is provided in Supplementary Text 1. All subjects provided written informed consent. The study was approved by the ethical committee of the Medical Faculty of the University of Cologne, Germany (ID: 23–1249) and conducted in accordance with the Declaration of Helsinki and its later amendments.

### 2.2 The picture‐based assessment of subjective deficits in cognition and activities of daily living (Pic‐ADL)

**FIGURE 2:**
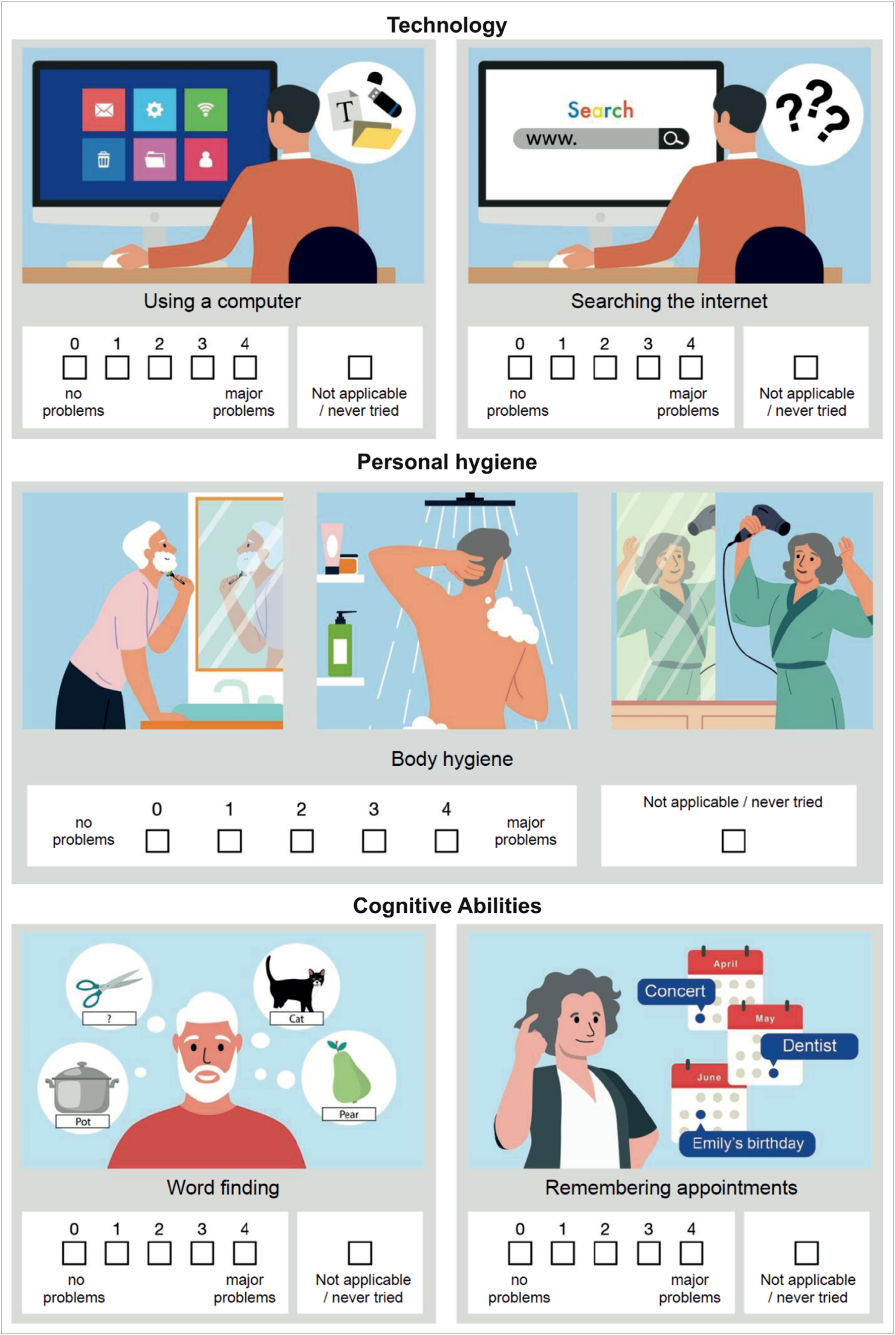
Example Items of the Pic‐ADL.

The Pic‐ADL is a predominantly picture‐based, self‐reported questionnaire designed to assess subjective difficulties in cognition and ADLs. It was developed collaboratively by neuropsychologists, neurolinguists, neurologists, and a graphic designer. Items are rated on a 5‐point Likert scale ranging from 0 (no problems) to 4 (major problems) (see example items in Figure 2).

The Pic‐ADL comprises 48 items across nine domains: Technology (9 items), Personal Hygiene (2 items), Healthcare (2 items), Household (4 items), Mobility (3 items), Finances (5 items), Social Life (2 items), Cognitive Abilities (17 items), and Other Issues (4 items). Completion time is ≈10–15 minutes. Total and domain scores are calculated as mean scores (sum divided by number of items completed), with a maximum of four points.

The Pic‐ADL is intended for individuals 40–90 years of age with suspected or diagnosed NCD, and it is freely accessible; all materials are provided in the Supplementary Material

The original questionnaire was developed in German; the translation procedure is described in the Supplementary Text 2.

#### 2.2.1 Item Selection

Item selection followed a multi‐step process. First, existing ADL and subjective cognitive complaint questionnaires were systematically reviewed, and relevant items were extracted. An interdisciplinary team evaluated these items for relevance, clarity, and graphical feasibility. Additional items were developed to capture domains insufficiently represented in existing instruments, particularly digital media use (e.g., smartphones, internet, social media) and financial management. All items were subsequently visualized by a graphic designer in collaboration with the research team. Illustrations depicted individuals representative of the target age range (40–90 years). The interdisciplinary team reviewed and iteratively refined the illustrations until consensus was reached on the final item set.

### 2.3 Instrument and Assessment

In addition to the Pic‐ADL, all participants completed a sociodemographic questionnaire, a neuropsychological test battery, and language‐based questionnaires. Controls completed a shortened version of the neuropsychological test battery used in patients.

#### 2.3.1 Neuropsychological test battery

The neuropsychological test battery consisted of the following tests:

1. Short Aphasia Check List^18^ to ensure adequate language comprehension for completing the validation instruments.
2. DemTect^20^ as a global cognitive screening.
3. Consortium to Establish a Registry for Alzheimer's Disease (CERAD)‐Plus battery^21^ including semantic and phonetic fluency tests, the 15–item Boston Naming test, the Mini‐Mental State Examination (MMSE), a Word List Learning/Recall/Recognition task, Figures Copy and Recall, and the Trail Making Test (TMT) A&B.
4. Digit Span Forward/Backward of the Wechsler Memory Scale—Revised (WMS‐R^22^) to evaluate memory and working memory.
5. Stroop Color‐Word‐Interference Test (Stroop^23^) as a measure of attention, processing speed, and inhibition.
6. Subtest Cube Analysis of the Visual Object and Space Perception Battery (VOSP^24^) to investigate visual perception.
7. Healthy controls completed only the Short Aphasia Check List, DemTect, TMT‐A&B, and the phonetic fluency task of the CERAD‐Plus test battery.

#### 2.3.2 Questionnaires

The following self‐reported questionnaires were included:

1) The Pic‐ADL was administered as a self‐report instrument. Participants were introduced to the response format using the first item as an example and completed the remaining items independently without assistance
2) Feedback Questionnaire on graphics, content, instructions, and completion time of the Pic‐ADL
3) Subjective Cognitive Decline Questionnaire (SCD‐Q^25^)
4) Perceived Deficits Questionnaire (PDQ^26^)
5) Bayer‐ADL (B‐ADL; self‐assessment^16^)
6) Amsterdam IADL Questionnaire Short version (A‐IADL‐Q)^27^, ^28^ for IADLs
7) BDI‐II^19^ for the intensity of depressive symptoms
8) The following informant‐based reports (if available) were included:
9) Informant Questionnaire on Cognitive Decline in the Elderly (IQCODE^29^)
10) B‐ADL (informant version)^16^

For more details on the questionnaires, see Supplementary Text 3. Patients could choose to complete the questionnaires either on‐site or at home (within 4 weeks), with the option to return them by post (mail).

The test–retest reliability control group completed the DemTect, TMT‐A&B, Pic‐ADL, and BDI‐II during the first assessment, and the DemTect and Pic‐ADL during the second retest assessment. The caption/no caption control group completed the same assessments as the control group.

### 2.4 Patient classification in SCD, Mild NCD, and major NCD

Cognitive impairment was determined according to the DSM‐5 framework,^30^ considering five cognitive domains: executive function, perceptual‐motor function, complex attention, learning and memory, and language (see Table S1 for test assignments^7^). Cognitive impairment was defined as present if^30^, ^31^:

  − One test score in any domain was ≥ 1.5 standard deviation (SD) below the normative mean, or
  − Two test scores within the same domain were ≥ 1 SD below the mean, or
  − Three test scores across domains were ≥ 1 SD below the mean.

ADL impairment was assessed using the B‐ADL (self‐ and informant‐rated), with an average score ≥3.0 on either rating indicating impairment.^32^

Based on objective cognitive performance and ADL functioning, patients were classified as mild or major NCD, defined as cognitive impairment without versus with ADL impairment. Patients without objective cognitive impairment were classified as having subjective cognitive decline (SCD), defined clinically by subjective cognitive difficulties documented during clinical history as the reason for presentation at the memory clinic.

### 2.5 Data analysis

Statistical analyses were conducted using SPSS Statistics, version 29.0,^33^ with a significance level of α = .05 (two‐tailed). A detailed description of the data analysis is available in the Supplementary Text 4.

Feasibility was evaluated by the proportion of missing item‐level data. Acceptability was assessed through (1) mean–median difference, (2) floor/ceiling effects, and (3) skewness. Group differences in feedback ratings between controls and NCD patients were examined using analysis of covariance (ANCOVA) with bootstrapping (age and education as covariates; α = .05; 5000 resamples, BCa intervals).

Internal consistency was examined for each domain and the total scale using (1) Cronbach's α and (2) corrected item‐total correlation. Test–retest reliability was examined using intraclass correlation coefficients (ICCs; two‐way random, absolute agreement, single measures) for the total and domain scores (Bonferroni‐adjusted). Measurement precision was estimated using the standard error of measurement (SEM), $\text{SEM}=SD\times \sqrt{1-r}$, where *r* denotes reliability (Cronbach's α).

Construct validity was evaluated using Spearman's rank correlations and Kendall's coefficient of concordance (*W*) to examine strength and direction of associations and agreement in rank ordering between Pic‐ADL and related questionnaires (A‐IADL‐Q, B‐ADL, SCD‐Q, PDQ). Associations with informant‐based measures (B‐ADL, IQCODE) were examined separately. Correlations/concordance were Bonferroni‐adjusted.

To examine the underlying structure of the Pic‐ADL, an exploratory factor analysis (EFA) was conducted on all items simultaneously using principal axis factoring. Sampling adequacy was assessed using the Kaiser–Meyer–Olkin (KMO) measure and Bartlett's test of sphericity. Factor retention was determined by eigenvalues >1 and scree plot inspection.

Known‐groups validity (controls vs mild NCD vs major NCD) was analyzed using ANCOVA with bootstrapping with age and education as covariates and Pic‐ADL total/domain scores as dependent variables (5000 resamples, BCa intervals). Differences in the DemTect were assessed using the same method (Bonferroni‐adjusted).

Diagnostic accuracy, as an indicator of criterion validity, was evaluated by receiver‐operating characteristic (ROC) analysis of the Pic‐ADL total score. The area under the curve (AUC) indicates discriminative ability. Optimal cutoffs distinguishing controls/mild NCD from major NCD were determined using the Youden index and the closest top‐left threshold. To characterize the severity of impairment on the Pic‑ADL total score, impairment levels were graded using cutoffs derived from the descriptive statistics of the control group. To more clearly distinguish between subjective cognitive complaints and functional ADL impairment, the total Pic‐ADL score was subdivided into two subscores: a functional ADL score and a cognitive score. ROC‐based procedures were applied to both subscores to estimate diagnostic accuracy and derive cutoff values.

## 3 RESULTS

### 3.1 Sample characteristics

According to the neuropsychological assessment, patients were classified as SCD (*n *= 8), mild NCD (*n *= 22), and major NCD (*n *= 40). Table 1 presents the sociodemographic characteristics and neuropsychological test performance of the control group and patient groups.

**TABLE 1:** Sociodemographic characteristics and neuropsychological test performance of the samples.

	Control group (*n *= 243)	All patients (*n *= 70)	SCD (*n *= 8)	Mild NCD (*n *= 22)	Major NCD (*n *= 40)
Age, years	56.96 ± 8.51 [40‐89]	69.99 ± 9.47 [43‐87]	65.88 ± 10.37 [47‐80]	68.95 ± 9.71 [43‐87]	71.38 ± 9.08 [51‐87]
Men/Women [%]	112/145 [43.6/56.4]	36/34 [51.4/48.6]	6/2 [75.0/25.0]	12/10 [54.5/45.5]	18/22 [45.0/55.0]
Education, years	16.93 ± 3.28 [6‐26]	14.38 ± 3.19 [7‐21]	15.50 ± 2.62 [12‐18]	15.59 ± 3.65 [7‐21]	13.49 ± 2.77 [9‐19]
Short Aphasia Check List, max. 40 points	38.99 ± 1.31 [34‐40]	35.81 ± 3.03 [26‐40]	37.25 ± 2.66 [34‐40]	36.89 ± 2.25 [32‐40]	34.93 ± 3.22 [26‐40]
DemTect, max. 18 points	16.97 ± 1.59 [13‐18]	12.67 ± 3.43 [5‐18]	15.75 ± 1.83 [13‐18]	13.50 ± 3.04 [7‐18]	11.60 ± 3.43 [5‐18]
CERAD‐Plus S‐words	16.91 ± 5.10 [4‐34]	11.69 ± 5.08 [1‐25]	16.75 ± 4.59 [11‐25]	12.18 ± 5.81 [1‐24]	10.40 ± 4.07 [3‐18]
CERAD‐Plus TMT‐A, max. 180 seconds	30.54 ± 11.55 [13‐94]	53.41 ± 24.01 [14‐179]	31.13 ± 11.18 [14‐50]	47.77 ± 18.62 [16‐98]	60.98 ± 25.15 [26‐179]
CERAD‐Plus TMT‐B, max. 300 seconds	71.91 ± 34.06 [20‐300]	159.73 ± 86.12 [36‐300]	83.00 ± 35.04 [36‐137]	131.86 ± 80.20 [50‐300]	190.40 ± 82.62 [47‐300]
BDI‐II, max. 63 points	3.19 ± 4.15 [0‐24]	9.29 ± 9.63 [0‐45]	6.88 ± 6.49 [3‐22]	6.14 ± 4.64 [0‐16]	11.50 ± 11.54 [0‐45]

*Note*: Values are reported as mean ± SD [range] unless otherwise indicated. Education (years) represents the total of years of schooling plus vocational and/or tertiary education. No participant self‐identified as non‐binary.

Abbreviations: BDI‐II, Beck Depression Inventory‐II; CERAD, Consortium to Establish a Registry for Alzheimer's Disease; NCD, neurocognitive disorder; SCD, subjective cognitive decline; SD, standard deviation; TMT‐A, Trail Making Test Part A; TMT‐B, Trail Making Test Part B.

### 3.2 Feasibility and Acceptability

All patients decided to complete the questionnaires at home and returned them by post (mail). Missing data for all items were minimal (0.3%–2.2%), indicating excellent feasibility. Results for acceptability and internal consistency in the combined sample of controls and neurological patients are displayed in Table 2. The difference between the mean and median of the Pic‐ADL total score (0.17 points, 4.3% of the maximum possible score) met acceptability criteria, as did all domain scores except for “Mobility” (0.42 points, 10.5%).

**TABLE 2:** Feasibility, acceptability, and internal consistency for the Pic‐ADL.

	Mean	Median	SD	Skewness	Minimum	Maximum	Floor effect (%)	Ceiling effect (%)	Cronbach's α	ICC (test–retest subsample)
Technology	0.52	0.22	0.76	2.21	0.00	4.00	30.2	0.3	.85	.58*
Personal Hygiene	0.11	0.00	0.36	3.64	0.00	2.00	89.1	0.0	.77	.33*
Healthcare	0.22	0.00	0.52	3.06	0.00	3.50	77.7	0.0	.71	.57*
Household	0.21	0.00	0.44	2.76	0.00	2.25	71.2	0.0	.76	.43*
Mobility	0.42	0.00	0.67	2.12	0.00	4.00	54.3	0.3	.70	.43*
Finances	0.13	0.00	0.39	4.41	0.00	3.00	77.9	0.0	.82	.55*
Social Life	0.29	0.00	0.60	2.84	0.00	4.00	71.8	0.3	.69	.50*
Cognitive Abilities	0.61	0.35	0.69	1.99	0.00	3.76	14.4	0.0	.95	.74*
Other Issues	0.85	0.75	0.81	1.22	0.00	4.00	20.2	0.3	.84	.62*
Pic‐ADL total	0.46	0.29	0.53	2.19	0.00	2.95	5.8	0.0	.95	.74*

*Note*: Total and domain scores are calculated relative to the number of items completed, with a maximum score of 4 points. Therefore, the Pic‐ADL total score represents an average score. Test–retest reliability was assessed in the test–retest reliability control group (n = 120) with total score and domain reliability assessed using the intraclass correlation coefficient (two‐way random, absolute agreement, single measures). After Bonferroni correction, *p*‐values below a significance level of α = .005 are considered significant.

Abbreviations: ICC, intraclass correlation coefficient; Pic‐ADL, Picture‐based assessment of subjective deficits in cognition and Activities of Daily Living; SD, standard deviation.

No notable floor (5.8%) or ceiling effects (0.0%) were observed for the total score. At the domain level, floor effects ranged from 14.4% to 89.1%, but were smaller among neurological patients (total score: 0.0%; domains: 1.4%–71.4%; see Table S2).

In the combined sample, the total score showed positive skewness (2.19), largely driven by low scores in the control group. In contrast, the patient group showed a more symmetrical distribution with acceptable skewness (0.79; see Table S2). The results for the control group are presented in Table S3.

Feedback ratings on graphics, content, instructions, and completion time were high across all participants, indicating good acceptability of the Pic‐ADL. Ratings did not differ significantly between controls and NCD patients (see Table S4), except for content ratings, which were slightly but significantly lower in the NCD group than in controls (controls: 9.36 ± 0.08 vs NCD: 8.81 ± 0.25, *p *= .023, η^2^ = 0.02).

Caption necessity was examined in a propensity score–matched control subsample (*n *= 14 pairs). No differences were found between Pic‐ADL versions with versus without captions for total or domain scores (all *p*’s > .05). Correlations with external measures remained statistically significant in both conditions (*p *< .05), indicating preserved convergent validity irrespective of caption use (see Supplementary Text 5).

### 3.3 Reliability

In the combined sample, item‐total corrected correlations met reliability criteria for nearly all items; only 1 of 48 items (sleep disturbances: *r *= .28) fell slightly below the standard. Cronbach's α values for the total scale indicated excellent internal consistency (α = 0.94–0.97 across groups). Across both the combined sample (Table 2) and the patient subsample (Table S2), only the “Social Life” domain showed α values < .70. Results for the control group are reported in Table S3. In the patient group, four items showed item‐total corrected correlations below the standard criterion (social contact: *r *= .23, remembering names: *r *= .24, recalling past events: *r *= .15; sleep disturbances: *r *= .12), whereas all other items ranged from *r *= .31 to .87.

Table 2 presents the test–retest reliability results, with a mean interval of 6.03 ± 0.76 weeks between assessments (range: 4.00–7.86 weeks). Test–retest reliability for the Pic‐ADL total score was high (ICC^1^, ^2^ = .74, 95% confidence interval [CI] .64– .81], *p *< .001).

Measurement precision indicated good reliability for the Pic‐ADL total score and most domains. Only three domains (“Healthcare,” “Mobility,” and “Social Life”) exceeded the 0.5 SD threshold, suggesting slightly lower precision in these areas (see Table S5).

### 3.4 Construct validity

Table S6 presents the correlations and concordance between the Pic‐ADL and established questionnaires assessing ADL (A‐IADL‐Q, B‐ADL self‐report) and subjective cognitive complaints (SCD‐Q, PDQ) for the combined sample. Results for controls and neurological patients are provided in Table S7.

In the combined sample, the Pic‐ADL total score correlated significantly with the reference instruments (*r_s_* = 0.42–0.77, *p *< .000025), indicating good convergent validity. Most domain scores also correlated moderately to strongly with existing measures (*r*_s _= 0.22–0.77, *p *< .00025). Concordance (Kendall's *W*) for the Pic‐ADL total score ranged from .58 to 1.00 (*p *< .000025), with most domains meeting the acceptable standard ( .56 to 1.00, *p *< .000025). Furthermore, the Pic‑ADL total score correlated significantly with the informant‑based B‑ADL (*r*_s _= .48, *p *< .000025) and IQCODE (*r*_s _= .47, *p *< .000025) and showed excellent agreement with both measures (B‑ADL: *W *= .91; IQCODE: *W *= 1.00, both *p*’s < .000025).

The “Other Issues” domain showed only moderate concordance with the B‐ADL or SCD‐Q but correlated significantly with the BDI‐II in the combined sample (*r*_s _= .60, *p *< .001). Nevertheless, its concordance with the BDI‐II was limited (*W *= .24, *p *< .001).

In addition, in the combined sample, the Pic‐ADL total score correlated significantly and moderately with the DemTect (*r*_s _= ‐.36), BDI‐II (*r*_s _= .53), age (*r*_s _= .46), and education (*r*_s _= ‐.28). The association with gender was negligible (see Table S8).

### 3.5 Dimensionality

Data were suitable for factor analysis (KMO = .86; Bartlett's *χ^2^* (1128) = 5786.50, *p *< .001). The first factor explained 35.0% of the variance and had a substantially higher eigenvalue than subsequent factors. Although the eigenvalue criterion suggested multiple factors, the scree plot revealed a clear break after the first factor, supporting a predominantly unidimensional structure.

An exploratory two‐factor solution suggested a broad distinction between items reflecting subjective cognitive complaints (e.g., memory, attention, and fatigue‐related symptoms) and items reflecting more complex IADLs (e.g., financial management and technology use). However, substantial cross‐loadings and a strong correlation between factors (*r *= .66) indicated that these dimensions were not clearly separable.

### 3.6 Known‐groups validity

Table 3 summarizes the known‐groups comparisons. The SCD group was excluded from these analyses due to its small sample size (*n *= 8). Across all Pic‐ADL domains—except “Social Life” and “Other Issues”—both controls and patients with mild NCD differed significantly from those with major NCD. For the “Cognitive Abilities” domain and the Pic‐ADL total score, all three groups differed significantly. Overall, total and domain scores increased with clinical severity (see Table 3).

**TABLE 3:** Known‐Groups Validity for the Pic‐ADL.

	Adjusted means ± standard error [95% CI bootstrapped]			ANCOVA		
	Control Group (*n *= 243)	Mild NCD (*n *= 22)	Major NCD (*n *= 40)	*F*(df)	*p*‐value	_p_η^2^
DemTect, max. 18 points	16.70 ± 0.12 [16.45‐16.94]	13.74 ± 0.61 [12.46‐14.92]	12.04 ± 0.63 [10.81‐13.26]	*F*(2, 300) = 69.62	<.001^1,2^	.46
Pic‐ADL						
Technology	0.38 ± 0.04 [0.31‐0.45]	0.47 ± 0.16 [0.19‐0.79]	1.31 ± 0.20 [0.93‐1.72]	*F*(2, 298) = 31.93	<.001^2,3^	.38
Personal Hygiene	0.05 ± 0.02 [0.02‐0.09]	0.10 ± 0.07 [‐0.02‐0.25]	0.45 ± 0.11 [0.26‐0.67]	*F*(2, 300) = 18.31	<.001^2,3^	.14
Healthcare	0.14 ± 0.03 [0.09‐0.19]	0.17 ± 0.06 [0.06‐0.30]	0.70 ± 0.15 [0.43‐0.99]	*F*(2, 297) = 16.92	<.001^2,3^	.11
Household	0.10 ± 0.02 [0.07‐0.14]	0.20 ± 0.10 [0.05‐0.39]	0.81 ± 0.13 [0.55‐1.07]	*F*(2, 296) = 50.61	<.001^2,3^	.37
Mobility	0.26 ± 0.03 [0.21‐0.32]	0.35 ± 0.10 [0.19‐0.55]	1.32 ± 0.17 [1.00‐1.67]	*F*(2, 300) = 50.11	<.001^2,3^	.35
Finances	0.05 ± 0.01 [0.03‐0.07]	0.08 ± 0.04 [0.01‐0.17]	0.63 ± 0.14 [0.37‐0.89]	*F*(2, 299) = 41.79	<.001^2,3^	.25
Social Life	0.15 ± 0.03 [0.09‐0.20]	0.50 ± 0.15 [0.25‐0.79]	1.03 ± 0.18 [0.70‐1.38]	*F*(2, 299) = 34.86	<.001^1,2^	.24
Cognitive Abilities	0.38 ± 0.03 [0.33‐0.43]	0.80 ± 0.09 [0.62‐0.98]	1.73 ± 0.16 [1.42‐2.03]	*F*(2, 300) = 93.03	<.001^1,2,3^	.49
Other Issues	0.70 ± 0.05 [0.62‐0.79]	1.03 ± 0.16 [0.73‐1.35]	1.52 ± 0.19 [1.14‐1.90]	*F*(2, 299) = 16.22	<.001^2^	.22
Pic‐ADL total, (max. 4 points)	0.30 ± 0.02 [0.27‐0.34]	0.54 ± 0.07 [0.42‐0.66]	1.28 ± 0.13 [1.03‐1.52]	*F*(2, 300) = 85.61	<.001^1,2,3^	.49

*Note*: Adjusted means from ANCOVA controlling for age and education. All models used bootstrapped confidence intervals (5000 resamples). After Bonferroni correction, *p*‐values below a significance level of α = .0045 are considered significant. ^1^Control Group vs Patients with mild NCD, ^2^Control Group vs Patients with major NCD, ^3^Patients with mild NCD vs Patients with major NCD. The SCD group was not included in the analysis due to the small sample size (*n *= 8). DemTect, higher scores indicate better performance. Pic‐ADL, higher scores indicate more problems.

Abbreviations: CI, confidence interval; NCD, neurocognitive disorder; Pic‐ADL, Picture‐based assessment of subjective deficits in cognition and Activities of Daily Living.

### 3.7 Criterion validity: Diagnostic performance

**FIGURE 3:**
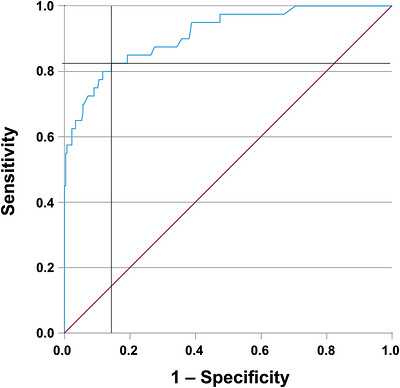
AUC of the Pic‐ADL Total Score for the Comparison Control Group/Patients with Mild NCD versus Patients with Major NCD. NOTE: The receiver‐operating characteristic curve (blue) illustrates the classification accuracy of the Pic‐ADL total score. The discriminatory power is demonstrated by AUC = .91. The diagonal red line represents chance‐level classification. The black reference lines mark the optimal threshold, corresponding to an 82.5% sensitivity and 85.7% specificity. NCD, neurocognitive disorder; Pic‐ADL, Picture‐based assessment of subjective deficits in cognition and Activities of Daily Living.

ROC curve analyses of the Pic‐ADL total score demonstrated high classification accuracy across all group comparisons (AUC = .79–.92; see Table S9). To distinguish controls and patients with mild NCD from patients with major NCD, the AUC was .91 (see Figure 3), indicating strong discriminatory power.

Based on both the Youden index and the closest top‐left approach, an optimal cutoff for the Pic‐ADL total score of 0.6 (sensitivity = .83, specificity = .86) was identified. Severity grading of ADL impairment on the Pic‑ADL was derived from the descriptive statistics of the control group (*M* = 0.29, *SD *= 0.27). Based on these values, mild impairment was defined as a total mean score ≥0.60, moderate as ≥0.90, and severe as ≥1.20. Concordance analyses with the B‑ADL self‑report supported this grading: participants with higher Pic‑ADL severity levels showed higher B‑ADL scores (mild: *M *= 1.94, *SD *= 0.79; moderate: *M *= 2.32, *SD *= 0.54; severe: *M *= 4.01, *SD *= 1.27), indicating increasing ADL impairment across both instruments.

ROC curve analyses demonstrated consistently high classification accuracy of the cognitive Pic‐ADL subscore across all group comparisons (AUC = .80–.92). As expected, the functional ADL subscore showed its strongest discriminative performance in comparisons involving major NCD, particularly in distinguishing major from mild NCD (AUC = .80) and from combined controls/mild NCD (AUC = .87; see Table S9)

Using the Youden index and the closest top‐left approach, optimal cutoff values were identified at 0.29 for the Pic‐ADL functional total score (sensitivity = .83, specificity = .77) and 0.76 for the Pic‐ADL cognitive total score (sensitivity = .85, specificity = .82).

## 4 DISCUSSION

The present study introduced the Pic‐ADL, the first predominantly picture‐based self‐report questionnaire designed to assess subjective cognitive and ADL complaints across NCD stages. The Pic‐ADL demonstrated strong psychometric properties, including high feasibility, reliability, validity, and high accuracy for distinguishing control subjects, mild NCD, and major NCD.

### 4.1 Comparison with previous ADL‐instruments

Compared with existing ADL instruments, the Pic‐ADL introduces several innovations. Traditional tools like the B‐ADL and Lawton IADL Scale capture digital activities only minimally (telephone use), whereas the Pic‑ADL includes a broader range of contemporary digital tasks. Although more recent tools such as the A‐IADL‐Q have expanded digital coverage, they remain language dependent. The picture‐based format of the Pic‐ADL substantially reduces linguistic demands and may therefore improve accessibility. It also offers more comprehensive coverage of financial activities, such as ATM withdrawals or card payment. In addition, other newer ADL tools, such as the Technology‑ADL Questionnaire,^34^ rely only on informant reports. Unlike most tools, which either combine functional and subjective cognitive complaints into a single scale^16^ or require separate instruments, the Pic‑ADL provides separate functional and cognitive scores.

### 4.2 Psychometric properties of the Pic‐ADL

Despite its minimal linguistic demands, the Pic‐ADL demonstrated strong psychometric performance. Feasibility was excellent, with minimal missing data and high user satisfaction. Versions with and without captions performed similarly, indicating that the images alone provide sufficient context. Captions were retained to reduce ambiguity, consistent with evidence that brief textual cues improve pictorial interpretation.^35^

Internal consistency and test–retest reliability were high for the total score, and construct validity was supported by substantial correlations with established ADL and subjective cognitive complaint instruments. As expected, associations for basic ADL domains with the A‐IADL‐Q, which focuses on complex instrumental activities, were weaker.^27^ The “Other Issues” domain correlated with the BDI‐II, consistent with its neuropsychiatric content.

The Pic‐ADL correlated more strongly with self‐report than informant‐based measures—an effect well documented in early cognitive decline.^11^, ^36^, ^37^ Reduced awareness of deficits in advanced NCD may lead to underestimation of impairment in self‐report measures, whereas informant reports are influenced by factors such as cohabitation, relationship, and educational background.^38^ Together, this supports integrating both perspectives, particularly in advanced disease stages.^39^

EFA findings suggest that the Pic‐ADL primarily reflects a strong general factor, indicating that subjective cognitive complaints and functional impairments are closely intertwined and may represent a unidimensional construct rather than distinct domains. In addition, the Pic‐ADL demonstrated high known‐group validity, with difficulty scores rising with diagnostic severity. In most domains, patients with major NCD reported greater difficulties than patients with mild NCD and controls. In the “Social Life” and “Other Issues” domains, both patient groups differed from controls but not from each other, consistent with evidence that social difficulties and neuropsychiatric symptoms often emerge early in the disease trajectory.^40^, ^41^ Furthermore, all three groups differed significantly in the “Cognitive Abilities” domain, reflecting the expected severity‐related gradient in cognitive difficulties.

Diagnostic accuracy was particularly strong when distinguishing major NCD from controls and mild NCD (AUC = .91). Even the diagnostically challenging differentiation between mild and major NCD showed high accuracy (AUC = .81) despite smaller group sizes and the narrower clinical distinction.^42^ Discrimination between controls and mild NCD was modest (AUC = .79), driven mainly by the cognitive subscore (AUC = .80), whereas the functional ADL subscore showed lower discrimination (AUC = .68). This aligns with the conceptual design of ADL‐based instruments, which aim to capture functional impairment; at the mild NCD stage, everyday functioning is often still largely preserved. Accordingly, the Pic‐ADL is intended for assessing functional impairment and supporting clinical staging along the NCD continuum rather than standalone screening for early cognitive decline.

Overall, the diagnostic performance of the Pic‑ADL is comparable to that of established ADL instruments. However, prior tools have predominantly demonstrated accuracy for broad distinctions, contrasting dementia with heterogeneous non‐dementia groups. For example, the A‐IADL‐Q reported an AUC of .75 when distinguishing dementia from a mixed non‐dementia sample (including MCI, SCD, psychiatric, and other neurological conditions),^43^ and Lawton's IADL scale achieved an AUC of .93 when separating dementia from a non‑dementia group including MCI and cognitively intact individuals.^44^ By contrast, the Pic‐ADL demonstrates strong performance not only for these broader distinctions but also for the clinically relevant, more demanding boundary between mild and major NCD.

For the Pic‐ADL total score, a cutoff of 0.6 points effectively differentiated individuals with and without ADL/subjective cognitive deficits. Furthermore, severity gradings (mild, moderate, severe) allow a more nuanced characterization of functional decline.

### 4.3 Strengths and limitations

This study has several strengths. First, the Pic‑ADL includes a digital media domain, capturing contemporary daily activities and the growing importance of electronic IADLs.^45^ Second, the predominantly picture‐based design may facilitate assessment of ADLs and subjective cognitive complaints when language‐based testing is challenging, potentially supporting more inclusive NCD diagnostics. The low linguistic demands also facilitate translation into other languages. Third, we provide a user‐friendly Excel file with scoring templates for the Pic‐ADL, enabling standardized and reproducible scoring. Fourth, the study benefited from a comprehensive neuropsychological test battery and multiple construct‐relevant questionnaires, allowing for robust validation.

Several limitations should be noted. The predominantly highly educated, ethnically homogeneous sample may limit generalizability. The different recruitment strategy for controls (via medical students’ personal networks) may have introduced selection bias and influenced the normative thresholds derived from this sample. Although analyses were adjusted for age and education, residual confounding cannot be excluded. Consequently, the derived thresholds should be interpreted with caution and validated in independent, more representative cohorts.

Test–retest reliability was assessed only in healthy controls, which may have resulted in restricted variance, possibly leading to artificially low SEM values. Future studies should evaluate test–retest reliability in clinically representative samples. Repeated exposure to identical picture‐based items may influence interpretation over time, particularly in longitudinal applications. Potential retest effects should be examined in future studies. Furthermore, several domain‐level scores showed lower reliability, suggesting limited stability. Accordingly, the total Pic‐ADL score may provide the most reliable measure of overall impairment, whereas domain‐specific scores should be interpreted cautiously.

Diagnostic classification relied partly on the B‐ADL, a widely used and validated instrument with established cutoff values for functional impairment.^32^ However, using an ADL‐based measure for both classification and outcome may have inflated diagnostic accuracy. Replication using alternative staging instruments, such as the CDR, is warranted to ensure a more independent reference standard.

Moreover, although the applied neuropsychological assessments covered the domains most relevant for diagnostic classification, the DSM‐5 domain of social cognition, an area with limited standardized assessment tools, was not explicitly assessed. Future studies may benefit from incorporating validated measures of social cognition to enable an even more comprehensive DSM‐5–aligned characterization.

Although the picture‐based format of the Pic‐ADL increases accessibility, most images depict White individuals, thereby limiting cultural representativeness; future versions should incorporate greater ethnic diversity. Furthermore, to date, the Pic‐ADL has only been validated in German. Although the predominantly visual format suggests that linguistic adaptations should be feasible, multilingual validation remains essential. Because captions are used, the Pic‐ADL is not entirely non‐verbal and still requires minimal language skills. Validation in individuals with language impairments was not feasible, as they could not complete the language‐dependent questionnaires. Future research should address this using adapted or nonverbal measures. However, combining pictorial stimuli with brief captions represents a pragmatic compromise, preserving the advantages of a largely language‑reduced format while supporting clarity and reliability of item interpretation.

Although the 48‑item Pic‑ADL was intended to comprehensively capture ADLs, high Cronbach's α values suggest some degree of item redundancy alongside strong internal consistency. A shortened version may retain comparable psychometric performance while reducing respondent burden. Further studies should explore item‑reduction strategies and evaluate whether a shortened version maintains validity and reliability, particularly in clinical and remote settings. In line with its conceptual focus on everyday digital media use, a digital version for smartphones and tablets is planned.

## 5 CONCLUSION

The Pic‐ADL represents the first predominantly picture‐based instrument to assess subjective cognitive complaints and everyday functional difficulties across NCD stages. In the present study, the Pic‐ADL demonstrated strong psychometric properties and high accuracy in distinguishing healthy adults and individuals with mild and major NCD.

Differentiating mild from major NCD is clinically relevant, as functional impairment directly influences diagnostic classification and subsequent care pathways. Individuals with mild NCD may still benefit from neuropsychological treatment, cognitive rehabilitation, and lifestyle‐based risk‐reduction strategies. In Alzheimer's disease, eligibility for recently FDA‐approved amyloid‐targeting therapies such as lecanemab^46^, ^47^ is currently restricted to early symptomatic stages with largely preserved basic ADLs. Conversely, documenting functional impairment is important, as it determines the need for additional support, higher long‐term care eligibility, and social service access. By capturing both subtle functional changes and more pronounced impairment, the Pic‐ADL may offer substantial practical value for treatment planning, prognostic assessment, and healthcare resource allocation. Its combination of traditional and modern ADL domains with minimal linguistic demands makes it a comprehensive and accessible tool for various clinical and research settings.

## AUTHOR CONTRIBUTION

**Isabell Ballasch**: Conceptualization, data curation, formal analysis, investigation, methodology, project administration, validation, visualization, writing – original draft, writing – review and editing. **Ronja Faßbender**: Data curation, investigation, validation, writing – review and editing. **Oezguer A. Onur**: Data curation, investigation, validation, writing – review and editing. **Sophia Felicitas Schuh**: Data curation, investigation, validation, writing – review and editing. **Nils Richter**: Data curation, investigation, validation, writing – review and editing. **Esra Kara**: Data curation, investigation, validation, writing – review and editing. **Anna Sauerbier**: Writing – review and editing. **Ulrike Lambotte**: Writing – review and editing. **Jutta Stahl**: Writing – review and editing. **Haidar S. Dafsari**: Writing – review and editing. **Gereon Rudolph Fink** Data curation, resources, writing – review and editing. **Elke Kalbe**: Resources, writing – review and editing. **Josef Kessler**: Conceptualization, data curation, funding acquisition, investigation, methodology, resources, validation, writing – review and editing. **Stefanie Theresa Jost**: Conceptualization, data curation, formal analysis, funding acquisition, investigation, methodology, project administration, resources, supervision, validation, visualization, writing – original draft, writing – review and editing.

## CONFLICT OF INTEREST STATEMENT

Isabell Ballasch was supported by the Brandau‐Laibach‐Foundation for the Pic‐ADL project. Furthermore, she was funded by the Prof. Klaus Thiemann Foundation.Ronja Faßbender reports no financial disclosures.Özgür A. Onur received lecture honoraria from Biogen, Eisai GmbH, Germany, Boston Scientific, Functional Neuromodulation, and Forum für medizinische Fortbildung FomF GmbH, and received consulting fees from Biogen GmbH, Germany.Sophia Schuh was supported by the Brandau‐Laibach‐Foundation for the Pic‐ADL project.Nils Richter has received funding from the Brandau‐Laibach‐Foundation.Esra Kara reports no financial disclosures.Anna Sauerbier was funded by the Gusyk program and the Advanced Cologne Clinician Scientist program of the Medical Faculty of the University of Cologne, and has received funding from the Prof. Klaus Thiemann Foundation, outside the submitted work.Ulrike Lambotte reports no financial disclosures.Jutta Stahl reports no financial disclosures.Haidar S. Dafsari's work was funded by the EU Joint Programme – Neurodegenerative Disease Research (JPND), the Prof. Klaus Thiemann Foundation in the German Society of Neurology, the Felgenhauer Foundation, and the Koeln Fortune program of the Medical Faculty of the University of Cologne, outside the submitted work; has received honoraria from Everpharma, Kyowa Kirin, Bial, Oruen, and Stadapharm, outside the submitted work; and serves as chair of a study group of the German Parkinson and Movement Disorders Society and steering group member of the Parkinson's Disease Non‐Motor Group (unpaid).Gereon R. Fink was funded by the Deutsche Forschungsgemeinschaft (DFG, German Research Foundation) – Project‐ID 431549029 – SFB 1451; and receives royalties from the publication of the books Funktionelle MRT in Psychiatrie und Neurologie, Neurologische Differentialdiagnose, SOP Neurologie, and Therapie‐Handbuch Neurologie; receives royalties from the publication of the neuropsychological tests KAS, NP‐KiSS, and Köpps; and received honoraria for speaking engagements from DGN and Forum für medizinische Fortbildung FomF GmbH.Elke Kalbe received honoraria from the companies EISAI GmbH, Germany, memodio GmbH, Germany, Desitin GmbH, Germany, and Prolog GmbH, Germany, all outside the submitted work.Josef Kessler was supported by the Brandau‐Laibach‐Foundation for the Pic‐ADL project. Furthermore, he received honoraria from the companies EISAI GmbH, Germany, Cogthera GmbH, Germany, and Prolog GmbH, Germany, all outside the submitted work.Stefanie T. Jost was supported by the Brandau‐Laibach‐Foundation for the Pic‐ADL project. Furthermore, she was funded by the Koeln Fortune Program/Faculty of Medicine, University of Cologne, by the Deutsche Forschungsgemeinschaft (DFG, German Research Foundation) – Project‐ID 431549029 – SFB 1451, and by the Prof. Klaus Thiemann Foundation, outside the submitted work. Author disclosures are available in the Supporting Information.

## CONSENT STATEMENT

*All subjects provided written informed consent. The privacy rights of all subjects have been observed. The study was approved by the ethical committee of the Medical Faculty of the University of Cologne, Germany (ID: 23‐1249) and conducted in accordance with the Declaration of Helsinki and its later amendments*.

## Supporting information



Supporting Information

Supporting Information

## Data Availability

The data used in this study are available from the corresponding authors upon reasonable request.
