# Supplementary material for: Development of a picture‐based tool for subjective deficits in cognition and ADLs
Source: Alzheimers Dement (Amst). 2026 Jun 29;18(3):e70413. doi: 10.1002/dad2.70413 (PMC13314551; doi:10.1002/dad2.70413)
Supplement: Supplementary file 1 — Supporting Information [file DAD2-18-e70413-s001.pdf]

SUPPLEMENTARY MATERIAL

Development of a picture-based tool for subjective deficits in cognition and ADL

Supplementary Material 1. Pic-ADL Questionnaire

Supplementary Text 1. Description of the Additional Control Samples

Supplementary Text 2. Translation of the Pic-ADL

Supplementary Text 3. Description of the Questionnaires

Supplementary Text 4. Data Analysis

Supplementary Text 5. Influence of Item Captions on Psychometric Properties

Supplementary Table 1. Cognitive Tests and Respective Cognitive Domain

Supplementary Table 2. Feasibility, Acceptability, and Internal Consistency of the Pic-ADL in Neurological Patients

Supplementary Table 3. Feasibility, Acceptability, and Internal Consistency of the Pic-ADL in the Control Group

Supplementary Table 4. Results of the Feedback Questionnaire of the Pic-ADL

Supplementary Table 5. Standard Error of Measurement of the Pic-ADL

Supplementary Table 6. Convergence and Agreement between the Pic-ADL and Existing Instruments (Spearman's Rank Correlation Coefficient and Kendall's Coefficient of Concordance) in the Combined Sample

Supplementary Table 7. Convergence and Agreement between the Pic-ADL and Existing Instruments (Spearman's Rank Correlation Coefficient and Kendall's Coefficient of Concordance) in the Control Group and Neurological Patients

Supplementary Table 8. Correlations of the Pic-ADL with Other Instruments and Demographic Variables

Supplementary Table 9. AUC of the Pic-ADL Total, Functional Total, and Cognitive Total Score

References

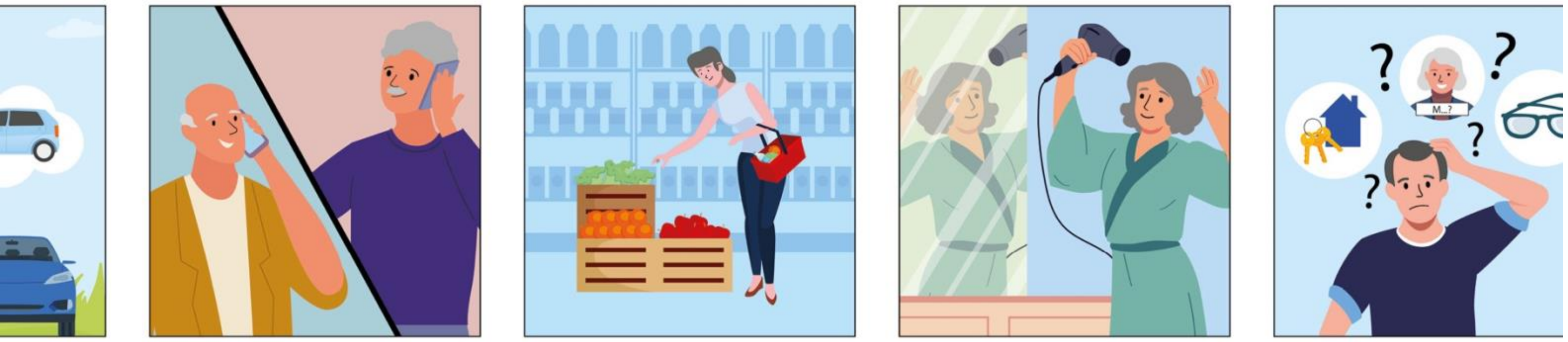

Picture-based assessment of subjective deficits in cognition and Activities of Daily Living
(Pic-ADL)

Instructions:
Please review each item and use the provided scale to indicate any difficulties you have experienced in your cognitive abilities and daily activities. Select the response that best reflects the extent of these difficulties over **the past four weeks.**
Kindly ensure that all questions are answered before submitting.

Technology

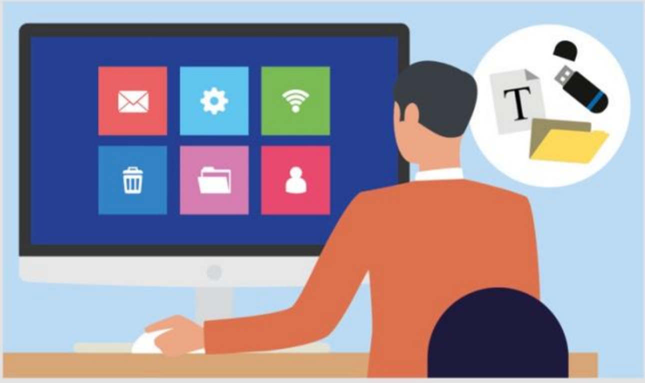

Using a computer

0	1	2	3	4	
☐	☐	☐	☐	☐	☐
no problems				major problems	Not applicable / never tried

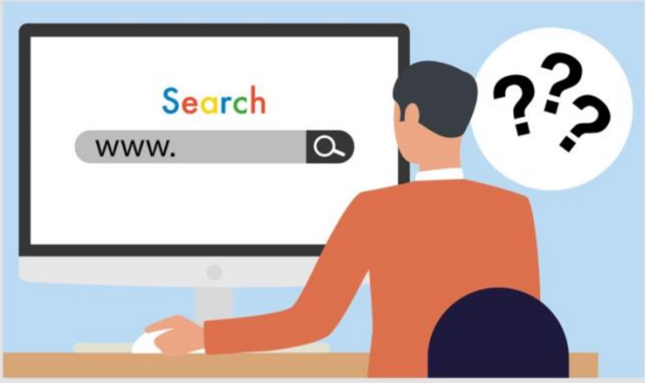

Searching the internet

0	1	2	3	4	
☐	☐	☐	☐	☐	☐
no problems				major problems	Not applicable / never tried

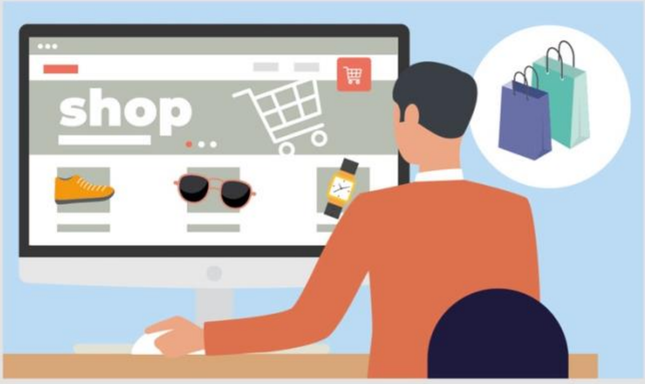

Shopping online

0	1	2	3	4	
☐	☐	☐	☐	☐	☐
no problems				major problems	Not applicable / never tried

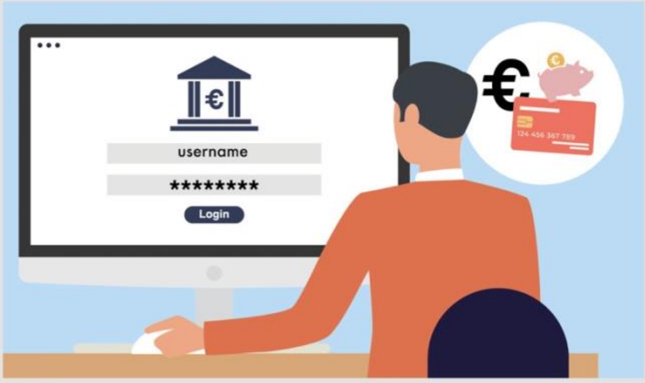

Online banking

0	1	2	3	4	
☐	☐	☐	☐	☐	☐
no problems				major problems	Not applicable / never tried

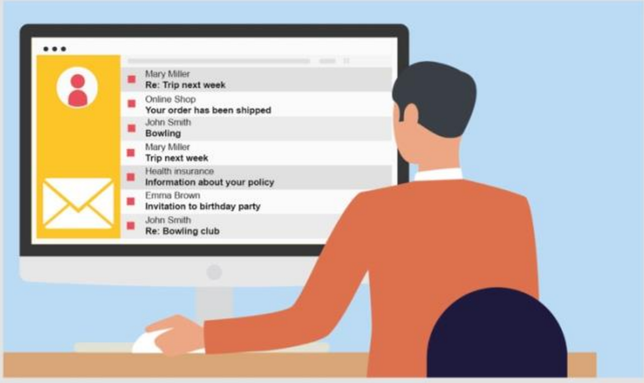

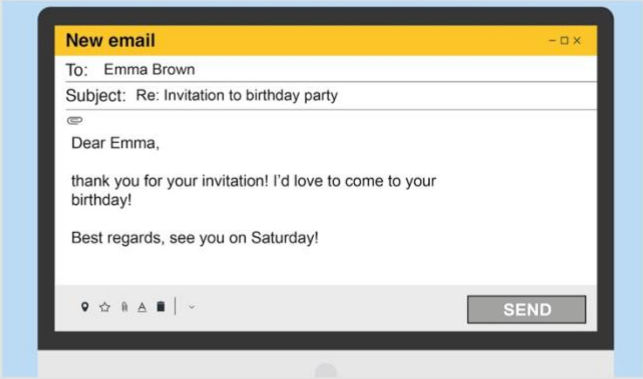

Using an email program

	0	1	2	3	4		Not applicable / never tried
no problems	☐	☐	☐	☐	☐	major problems	☐

Technology

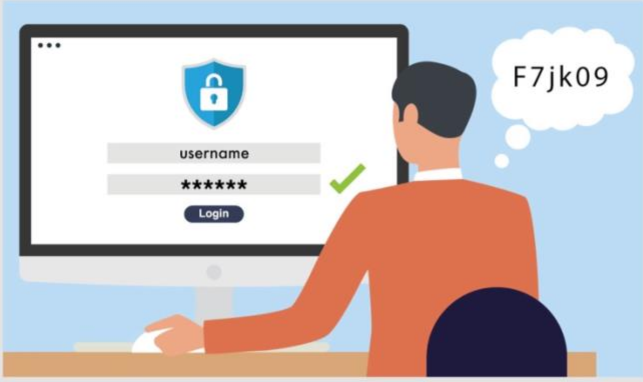

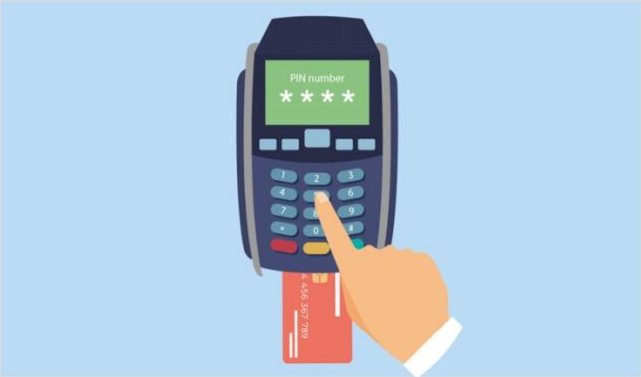

Remembering passwords and PIN codes

	0	1	2	3	4	
no problems	☐	☐	☐	☐	☐	major problems

Not applicable / never tried ☐

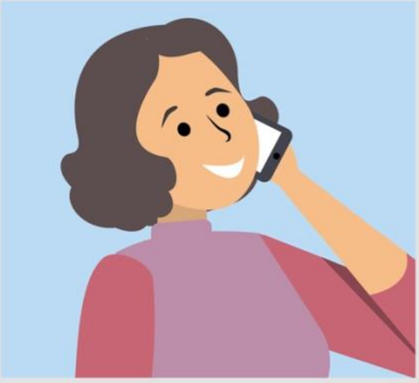

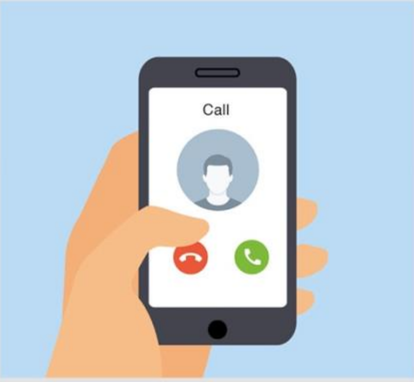

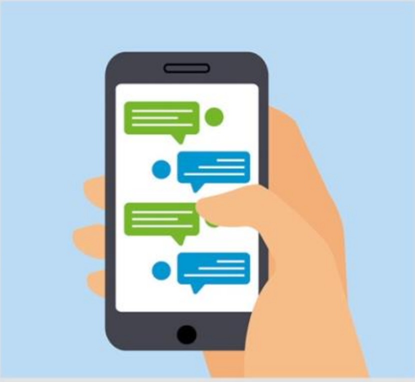

Using a mobile phone

	0	1	2	3	4	
no problems	☐	☐	☐	☐	☐	major problems

Not applicable / never tried ☐

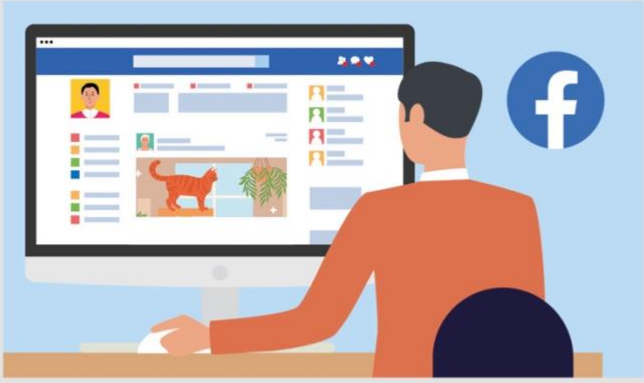

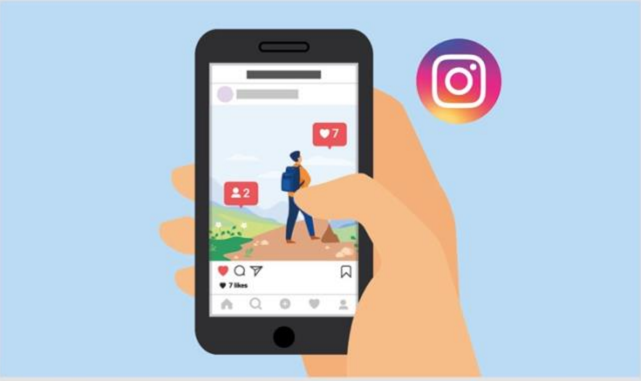

Using social media

	0	1	2	3	4	
no problems	☐	☐	☐	☐	☐	major problems

Not applicable / never tried ☐

Technology

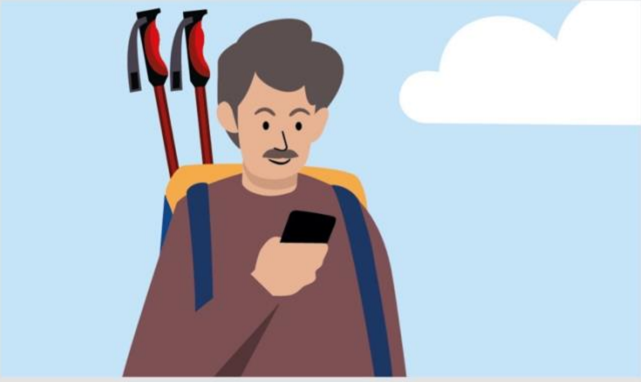

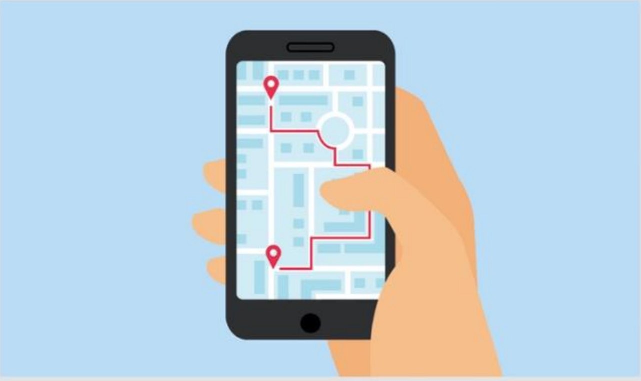

Using online navigation

no problems	0 ☐	1 ☐	2 ☐	3 ☐	4 ☐	major problems	Not applicable / never tried ☐

Personal Hygiene

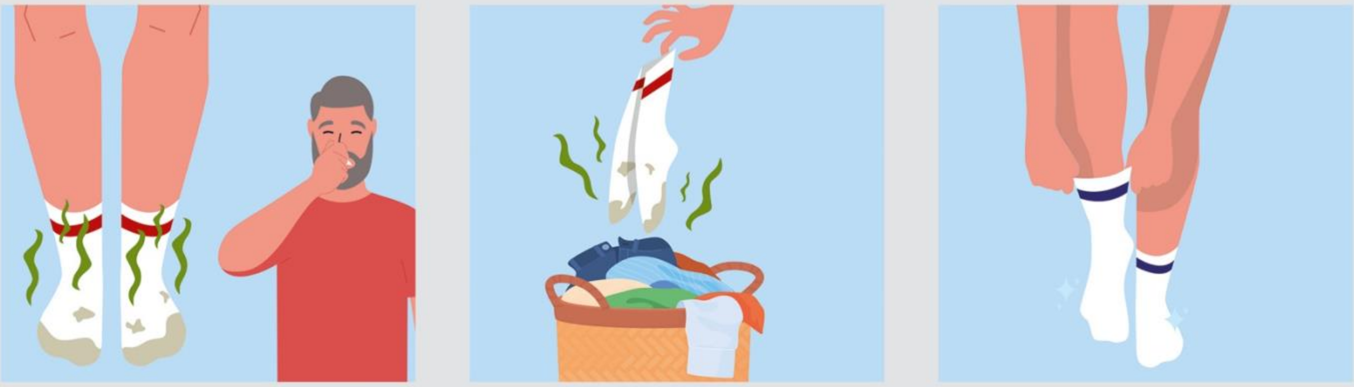

Putting on clean clothes

no problems	0 ☐	1 ☐	2 ☐	3 ☐	4 ☐	major problems

Not applicable / never tried ☐

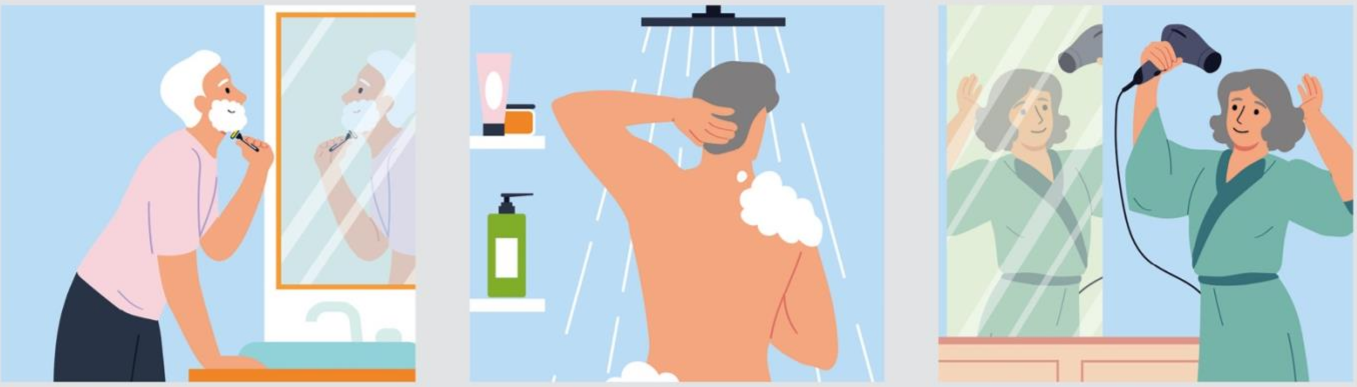

Body hygiene

no problems	0 ☐	1 ☐	2 ☐	3 ☐	4 ☐	major problems

Not applicable / never tried ☐

Healthcare

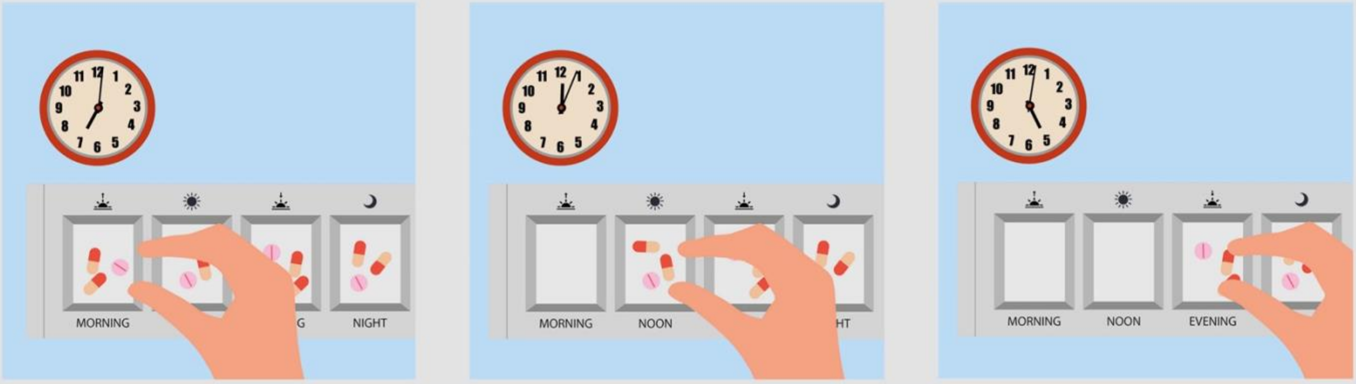

Taking medication

no problems	0	1	2	3	4	major problems	Not applicable / never tried
	☐	☐	☐	☐	☐		☐

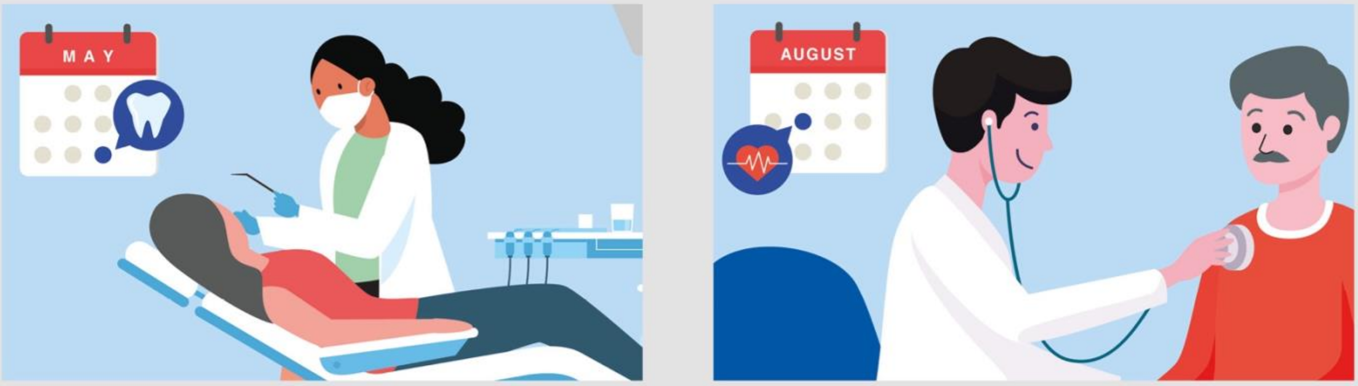

Regular doctor visits

no problems	0	1	2	3	4	major problems	Not applicable / never tried
	☐	☐	☐	☐	☐		☐

Household

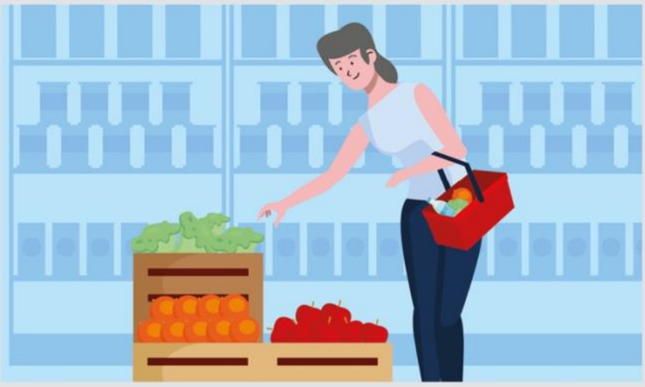

Shopping

0 1 2 3 4
☐ ☐ ☐ ☐ ☐
no problems … major problems

☐ Not applicable / never tried

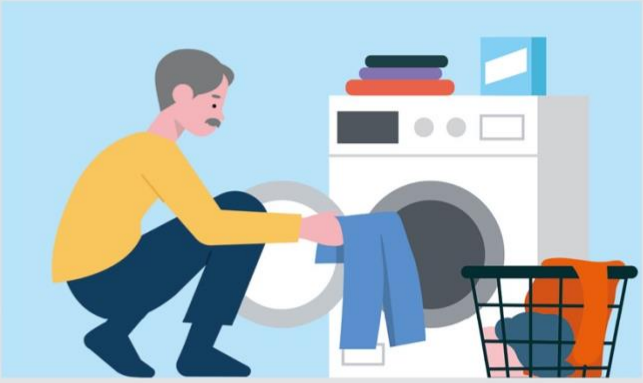

Doing laundry

0 1 2 3 4
☐ ☐ ☐ ☐ ☐
no problems … major problems

☐ Not applicable / never tried

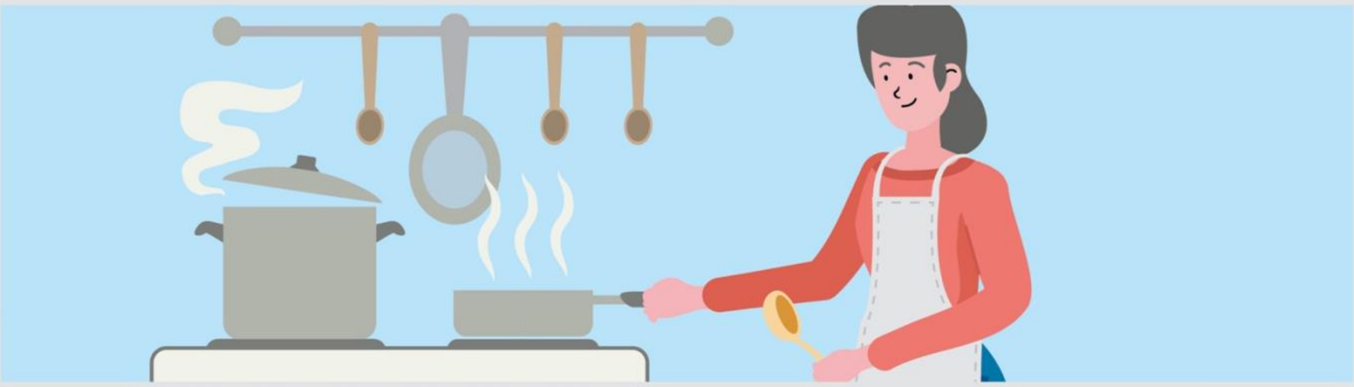

Cooking

no problems ☐ 0 ☐ 1 ☐ 2 ☐ 3 ☐ 4 major problems

Not applicable / never tried ☐

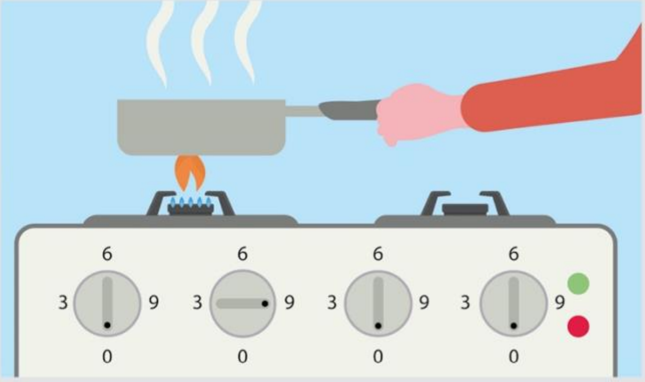

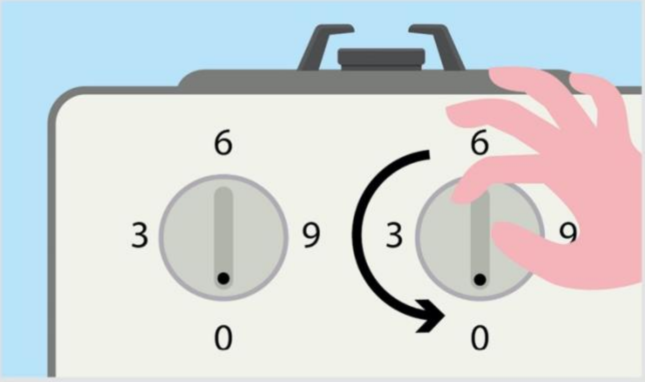

Turning off the stove after use

no problems ☐ 0 ☐ 1 ☐ 2 ☐ 3 ☐ 4 major problems

Not applicable / never tried ☐

Mobility

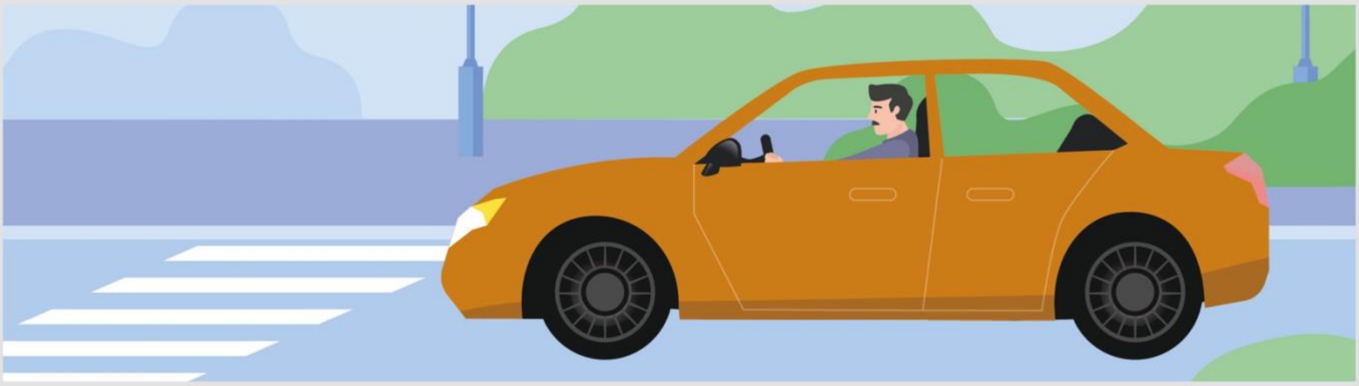

Driving a car

	0	1	2	3	4		Not applicable / never tried
no problems	☐	☐	☐	☐	☐	major problems	☐

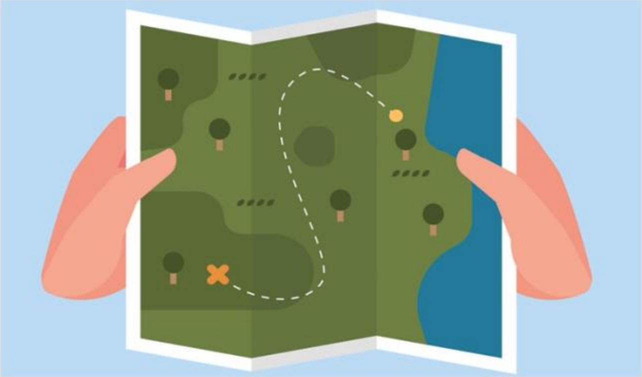

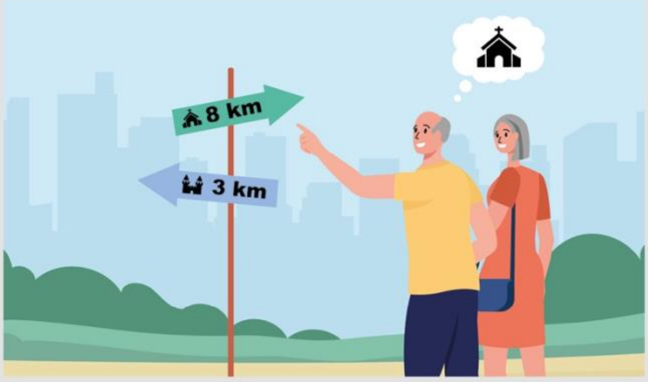

Spatial orientation

	0	1	2	3	4		Not applicable / never tried
no problems	☐	☐	☐	☐	☐	major problems	☐

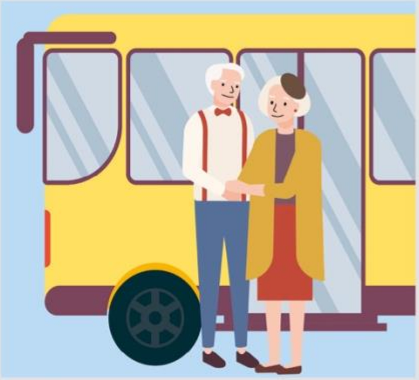

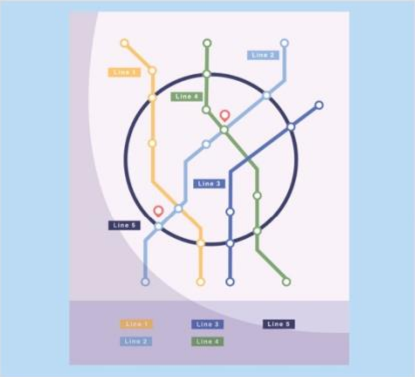

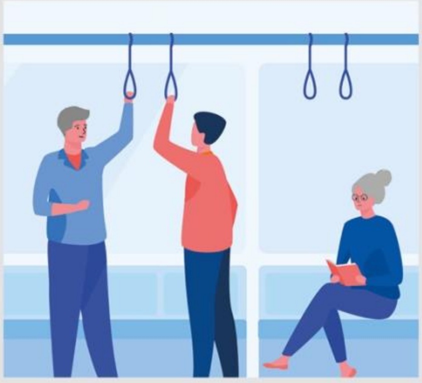

Using public transportation

	0	1	2	3	4		Not applicable / never tried
no problems	☐	☐	☐	☐	☐	major problems	☐

Finances

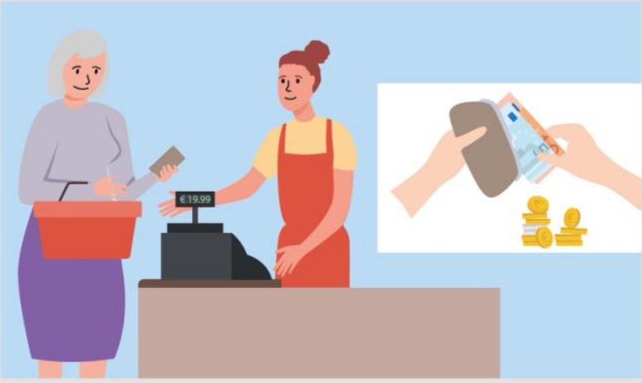

Paying with cash

0 ☐ 1 ☐ 2 ☐ 3 ☐ 4 ☐

no problems … major problems

☐ Not applicable / never tried

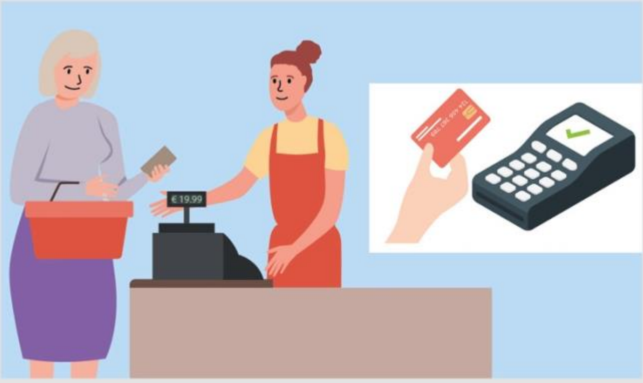

Paying by card

0 ☐ 1 ☐ 2 ☐ 3 ☐ 4 ☐

no problems … major problems

☐ Not applicable / never tried

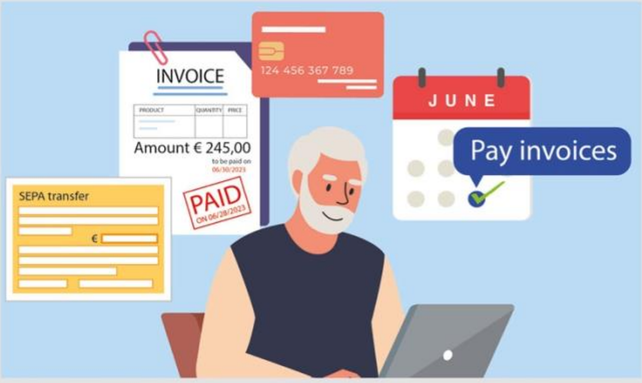

Paying bills on time

0 ☐ 1 ☐ 2 ☐ 3 ☐ 4 ☐

no problems … major problems

☐ Not applicable / never tried

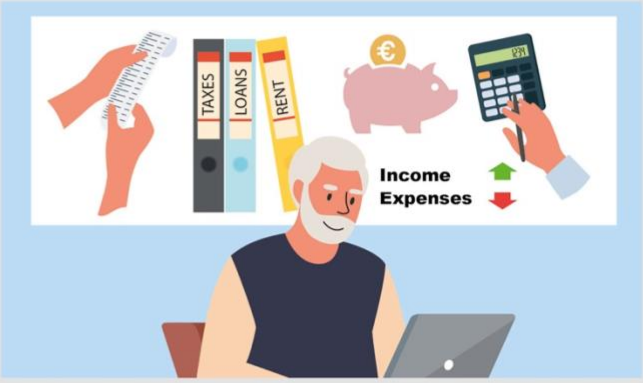

Managing personal finances

0 ☐ 1 ☐ 2 ☐ 3 ☐ 4 ☐

no problems … major problems

☐ Not applicable / never tried

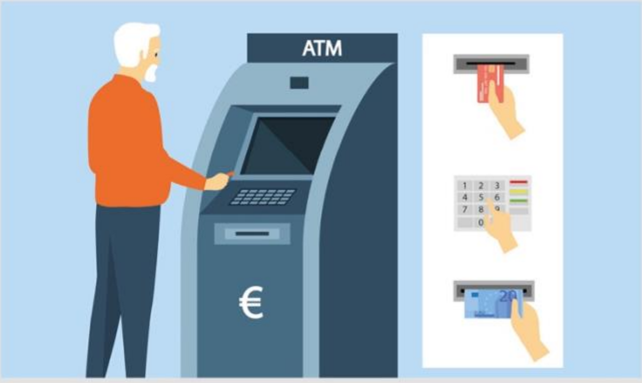

Withdrawing money from an ATM

0 ☐ 1 ☐ 2 ☐ 3 ☐ 4 ☐

no problems … major problems

☐ Not applicable / never tried

Social Life

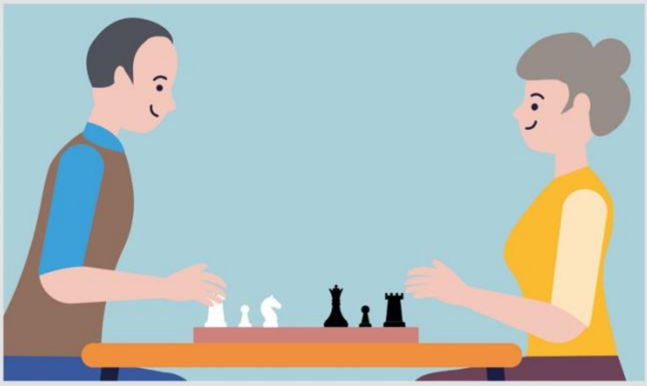

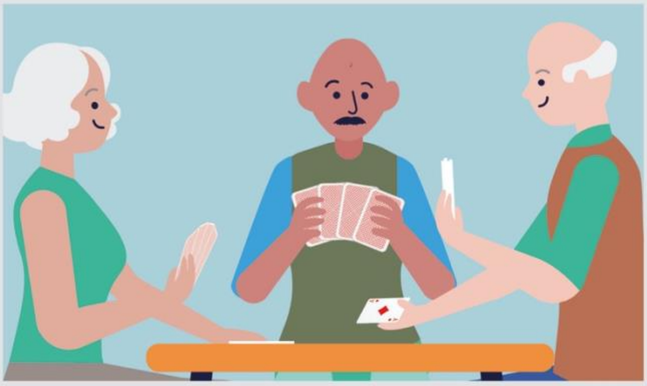

Playing board games / cards

	0	1	2	3	4		Not applicable / never tried
no problems	☐	☐	☐	☐	☐	major problems	☐

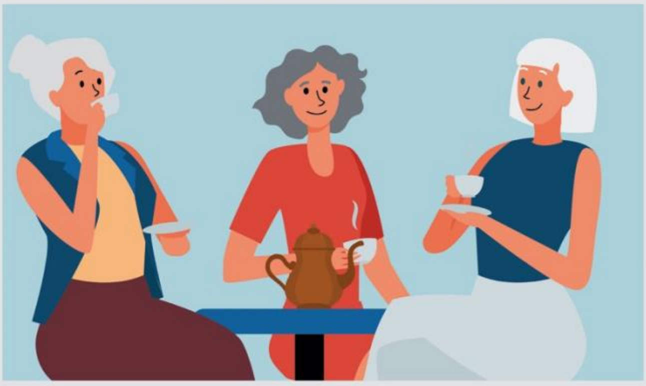

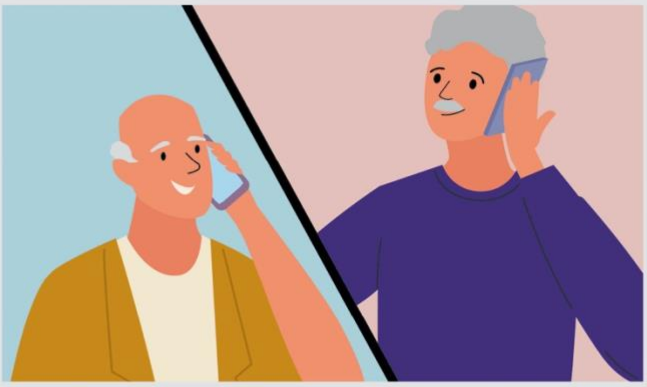

Social contacts

	0	1	2	3	4		Not applicable / never tried
no problems	☐	☐	☐	☐	☐	major problems	☐

Cognitive Abilities

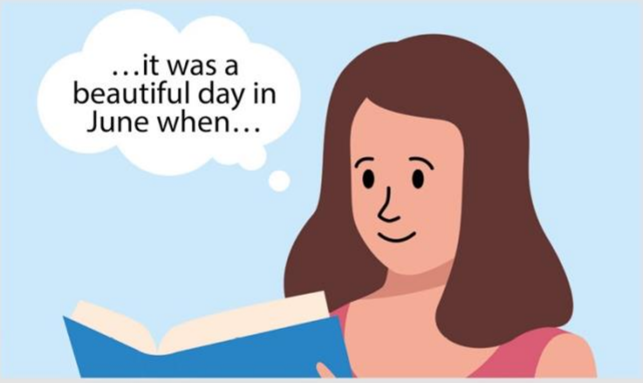

Reading

0	1	2	3	4	
☐	☐	☐	☐	☐	☐
no problems				major problems	Not applicable / never tried

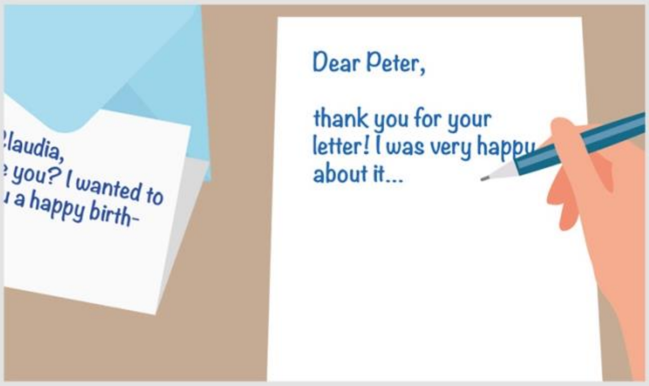

Writing

0	1	2	3	4	
☐	☐	☐	☐	☐	☐
no problems				major problems	Not applicable / never tried

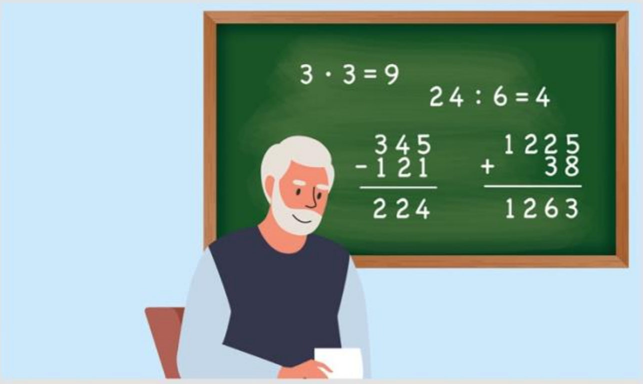

Calculating

0	1	2	3	4	
☐	☐	☐	☐	☐	☐
no problems				major problems	Not applicable / never tried

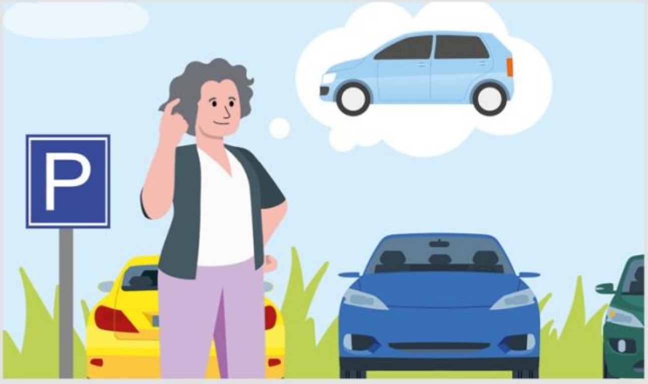

To find car in parking lot

0	1	2	3	4	
☐	☐	☐	☐	☐	☐
no problems				major problems	Not applicable / never tried

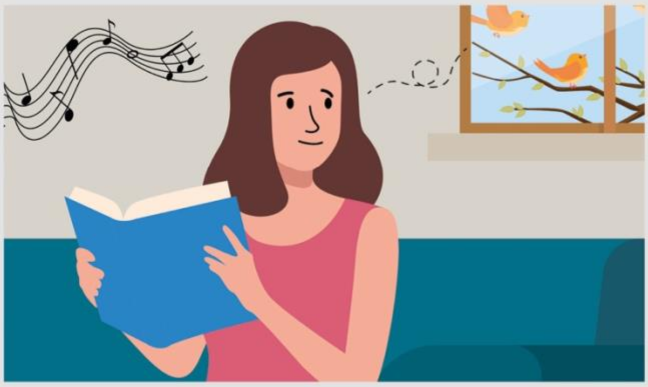

Get distracted

0	1	2	3	4	
☐	☐	☐	☐	☐	☐
no problems				major problems	Not applicable / never tried

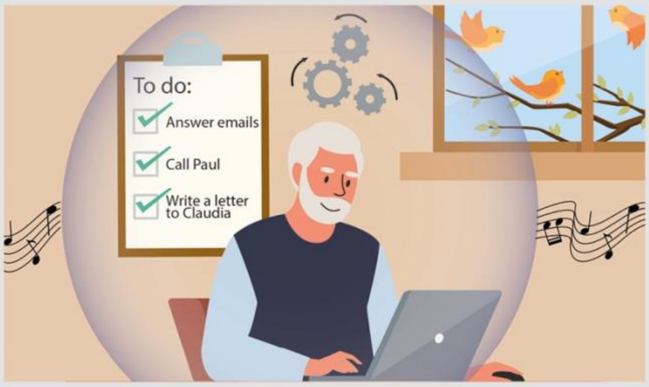

Concentration

0	1	2	3	4	
☐	☐	☐	☐	☐	☐
no problems				major problems	Not applicable / never tried

Cognitive Abilities

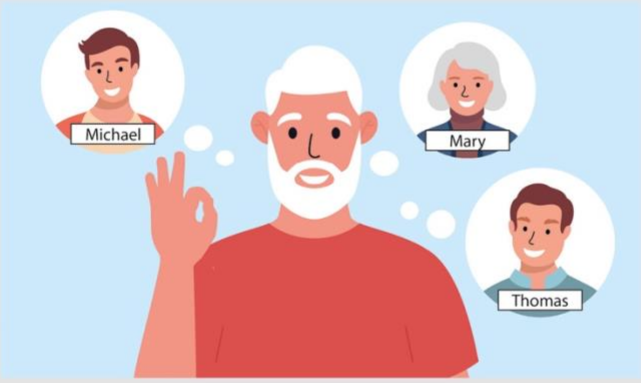

Remembering names

0	1	2	3	4	
☐	☐	☐	☐	☐	☐
no problems				major problems	Not applicable / never tried

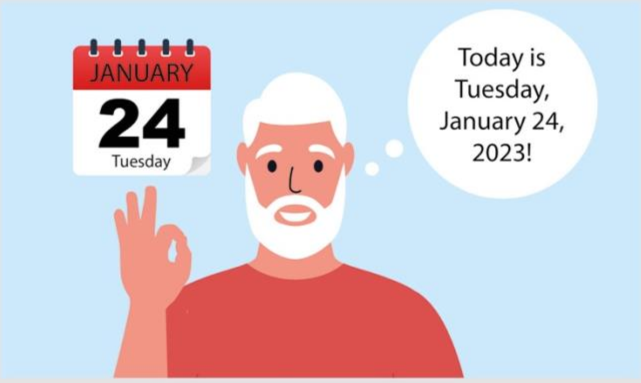

Awareness of time

0	1	2	3	4	
☐	☐	☐	☐	☐	☐
no problems				major problems	Not applicable / never tried

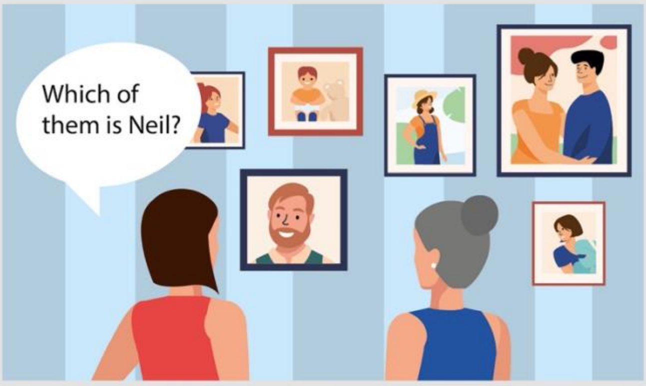

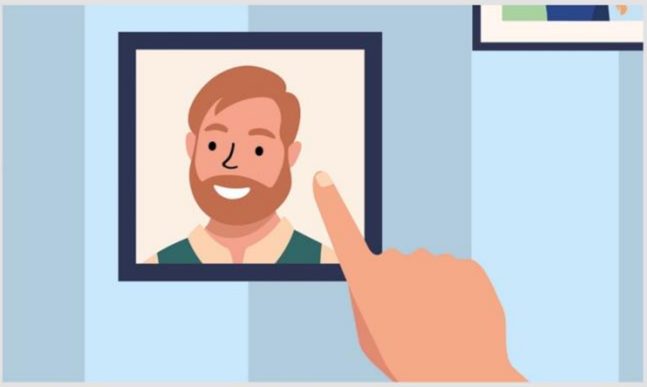

Remembering faces

	0	1	2	3	4		Not applicable / never tried
no problems	☐	☐	☐	☐	☐	major problems	☐

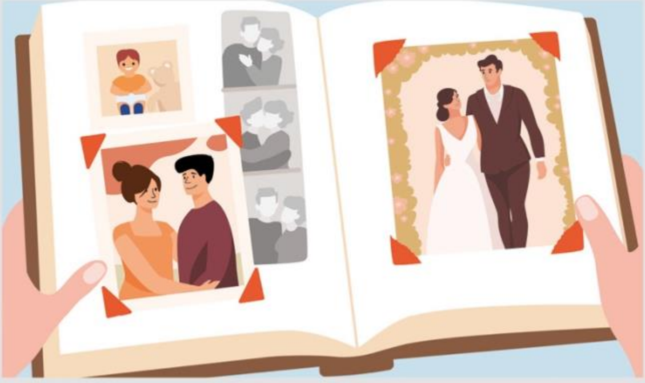

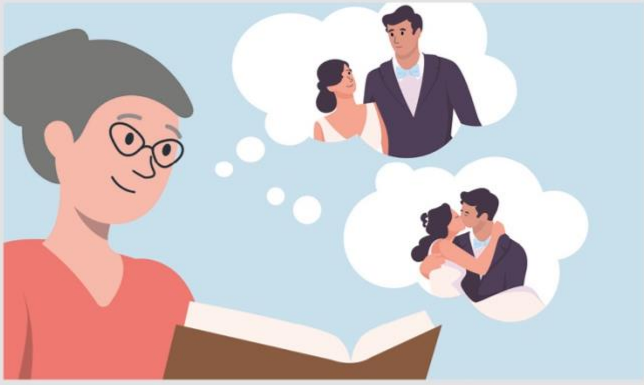

Recalling past events

	0	1	2	3	4		Not applicable / never tried
no problems	☐	☐	☐	☐	☐	major problems	☐

Cognitive Abilities

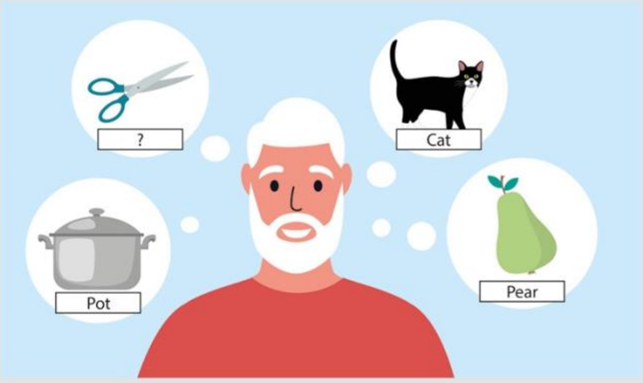

Word finding

0	1	2	3	4	
☐	☐	☐	☐	☐	☐
no problems				major problems	Not applicable / never tried

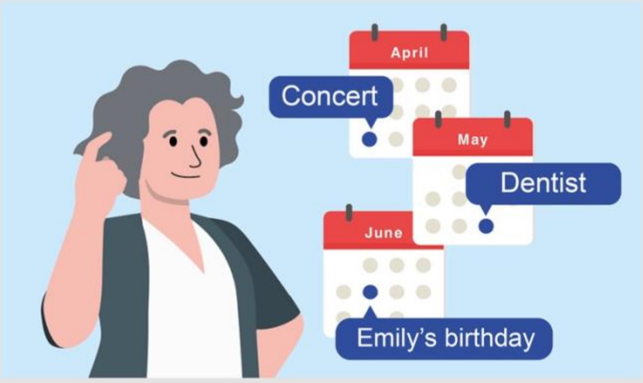

Remembering appointments

0	1	2	3	4	
☐	☐	☐	☐	☐	☐
no problems				major problems	Not applicable / never tried

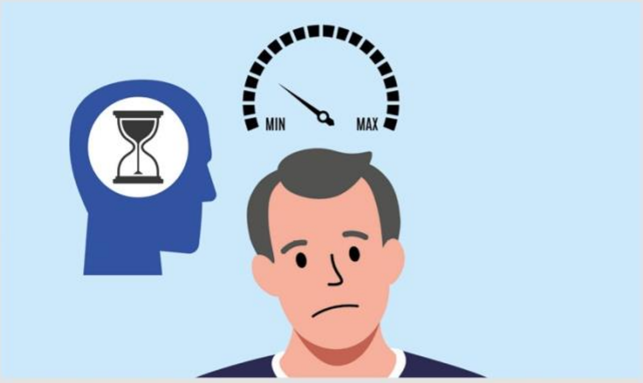

Mental slowing

0	1	2	3	4	
☐	☐	☐	☐	☐	☐
no problems				major problems	Not applicable / never tried

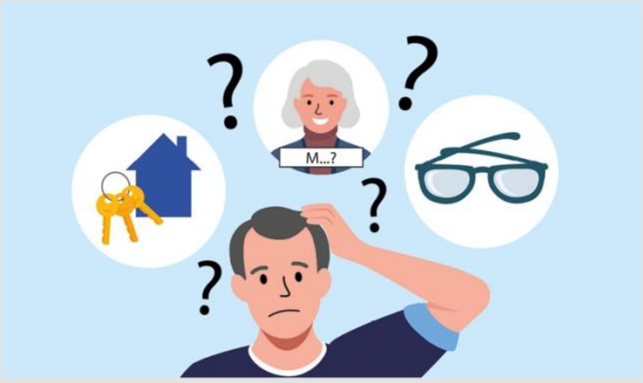

Memory

0	1	2	3	4	
☐	☐	☐	☐	☐	☐
no problems				major problems	Not applicable / never tried

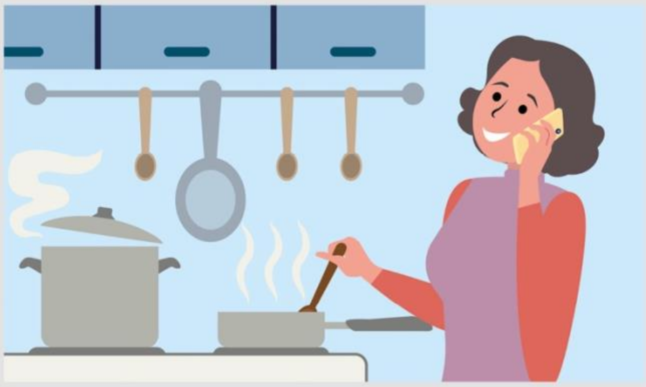

Multitasking

0	1	2	3	4	
☐	☐	☐	☐	☐	☐
no problems				major problems	Not applicable / never tried

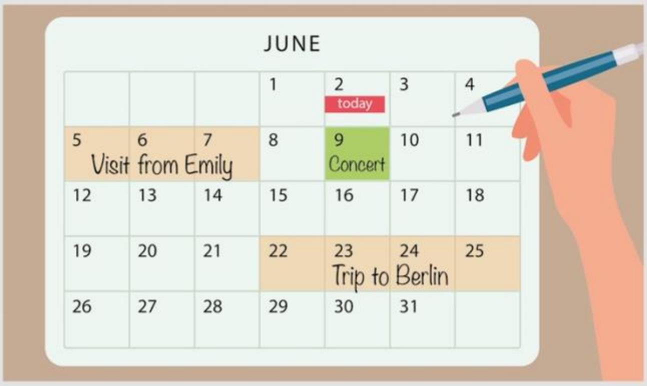

Planning ahead

0	1	2	3	4	
☐	☐	☐	☐	☐	☐
no problems				major problems	Not applicable / never tried

Cognitive Abilities

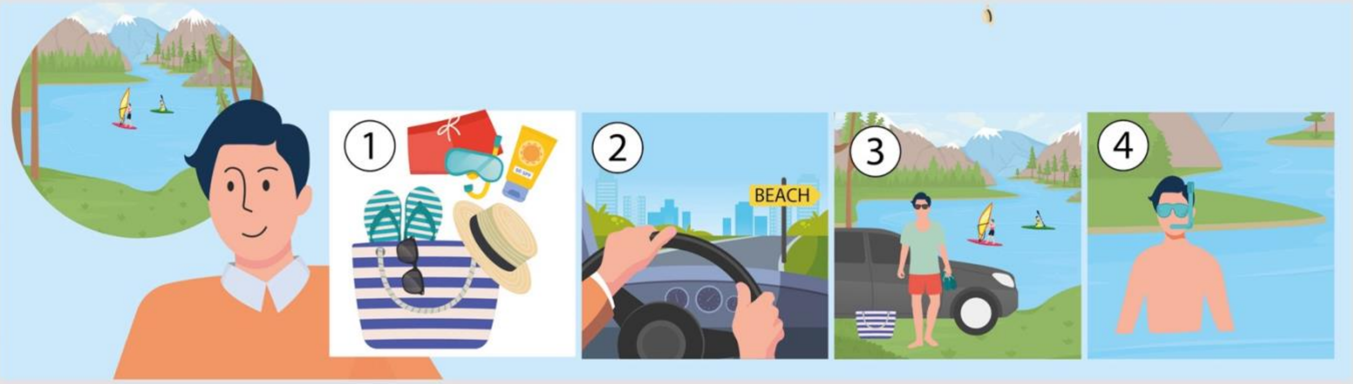

Activity planning

no problems	0	1	2	3	4	major problems
	☐	☐	☐	☐	☐	

Not applicable / never tried

☐

Other Issues

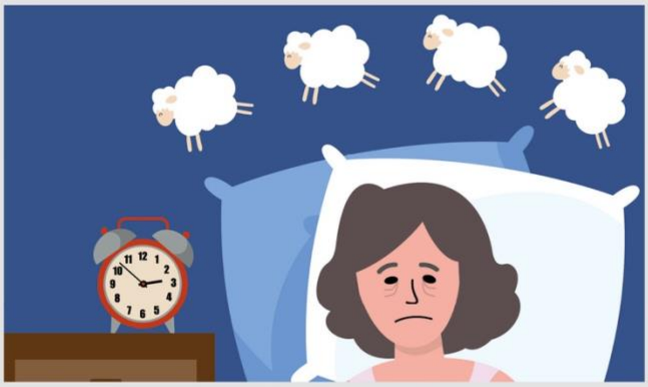

Sleep problems

0	1	2	3	4	
☐	☐	☐	☐	☐	☐
no problems				major problems	Not applicable / never tried

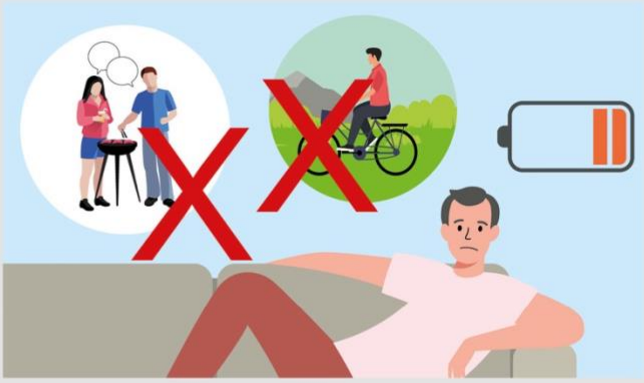

Apathy / Low drive

0	1	2	3	4	
☐	☐	☐	☐	☐	☐
no problems				major problems	Not applicable / never tried

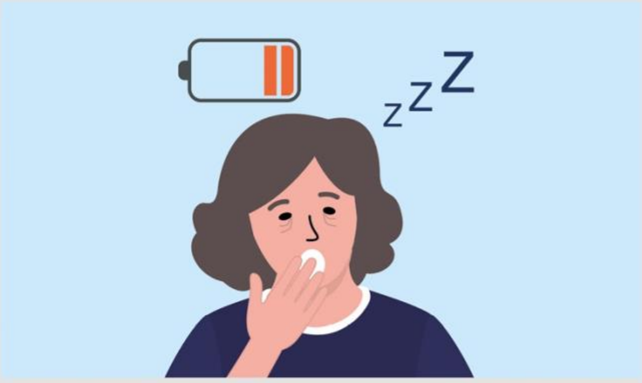

Fatigue / Tiredness

0	1	2	3	4	
☐	☐	☐	☐	☐	☐
no problems				major problems	Not applicable / never tried

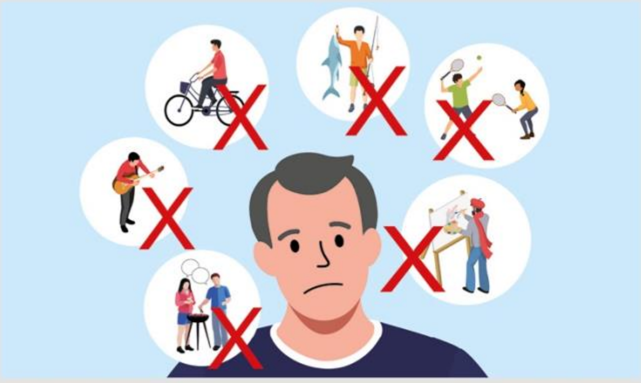

Loss of interest

0	1	2	3	4	
☐	☐	☐	☐	☐	☐
no problems				major problems	Not applicable / never tried

Supplementary Text 1. Description of the Additional Control Samples

Test-retest reliability group: *n=120* control participants assessed between May and October 2024 to assess test-retest reliability. For the retest evaluation, all participants were asked to complete the Pic-ADL a second time six weeks after the initial completion. The retest assessment was conducted in person during home visits. The test-retest reliability control group (*n*=120) had a mean age of 59.46 ± 12.26 years, an average of 16.25 ± 3.86 years of education, and consisted of 78 women (65.0%) and 42 men (35.0%).

Caption/no caption control group: *n*=40 control participants were recruited between October and November 2023 to examine the impact of picture captions on the psychometric properties of the Pic-ADL. This group was equally split between versions with and without captions. Both the caption and no-caption groups comprised 20 participants each. The caption group had a mean age of 61.75 ± 10.18 years, mean education of 16.05 ± 3.90 years, and included 12 women (60.0%) and 8 men (40.0%), while the no-caption group had a mean age of 56.60 ± 9.30 years, mean education of 16.85 ± 3.42 years, with 10 women (50.0%) and 10 men (50.0%).

Supplementary Text 2. Translation of the Pic-ADL

To ensure linguistic and conceptual equivalence for the English version, a multi-step translation and back-translation procedure was conducted following established guidelines for cross-cultural adaptation of assessment instruments [1]. First, five bilingual translators (native in German and English) independently translated the original German version into English. Their translations were reviewed and synthesized into a coherent preliminary English version by a native English speaker residing in the United States. This English version was back-translated into German by two bilingual translators (native in German and English). The instrument developers compared the back-translated version with the original questionnaire to identify and resolve discrepancies in meaning, terminology, or cultural relevance. Minor wording adjustments were made to ensure conceptual consistency and clarity across both language versions.

Supplementary Text 3. Description of the Questionnaires

The following self-reported questionnaires were included:

1) Pic-ADL.
2) Feedback Questionnaire on graphics, content, instructions, and completion time of the Pic-ADL.
3) Subjective Cognitive Decline Questionnaire (SCD-Q) assesses cognitive complaints across six domains: memory, attention, language, executive functions, visuospatial abilities, and social cognition. For each domain, participants first indicate whether they perceive a decline (yes/no), then rate their concern (3-point Likert scale: ‘no‘=0, ‘sometimes‘=1, ‘yes‘=2), and finally evaluate specific everyday difficulties (4-point Likert scale: ‘never‘=0 to ‘always‘=3).
4) Perceived Deficits Questionnaire (PDQ) measures subjective cognitive complaints across four domains: attention, retrospective memory, prospective memory, and planning/organization. It comprises 20 items, each rated on a 5-point scale ranging from ‘never‘ (0) to ‘almost always‘ (4).
5) Bayer-ADL (B-ADL) was used as a self-assessment ADL scale. It includes 25 items asking how often difficulties occur in specific everyday activities, ranging from one to 10.
6) A-IADL-Q-Short version evaluates IADL using 30 items. Each item is rated on a 5-point scale and grouped into eight subcategories: household, administration, work, computer use, leisure, devices, transportation, and other activities.
7) BDI-II is a 21-item self-administered inventory designed to measure the intensity of depressive symptoms on a 4-point scale.

The following informant-based reports (if available) were included:

8) Informant Questionnaire on Cognitive Decline in the Elderly (IQCODE): seven items related to memory ability and intellectual functions are rated on a 5-point scale from 1 (‘much improved‘) to 5 (‘much worse‘).
9) B-ADL (informant version).

Supplementary Text 4. Data Analysis

Feasibility and Acceptability

Feasibility was evaluated by the proportion of missing item-level data (acceptable if <5% [2]). Acceptability was assessed through (i) mean-median difference (satisfactory if ≤10% of the maximum possible score or ≤0.40 points [3]), (ii) floor/ceiling effects (satisfactory if ≤15% of respondents achieve the minimum or maximum possible score [4]), and (iii) skewness (acceptable if between -1 and +1, indicating approximately symmetric score distributions [5]).

Additionally, the Feedback Questionnaire was analyzed descriptively. Group differences in feedback ratings between controls and NCD patients were examined using ANCOVA (age and education as covariates; α=.05). Due to violations of normality and homogeneity of variance, analyses used bootstrapping (5,000 resamples, BCa intervals).

Reliability

Internal consistency was examined for each domain and the total scale using (i) Cronbach's α (α≥.70 [6]) and (ii) corrected item-total correlation (*r*≥.30 [5]).

Test-retest reliability was examined using intraclass correlation coefficients (ICC; two-way random, absolute agreement, single measures) for the total and domain scores (ICC≥.70 [7, 8]). Bonferroni corrections were applied α=.005 (α=.05/10).

Measurement precision was estimated using the standard error of measurement (SEM), (SEM)=SD x $\sqrt{1-r}$, where *r* denotes reliability (Cronbach's α). SEM provides an estimate of the precision of individual scores by quantifying the expected variability of an observed score around the true score. SEM was included to complement reliability indices by offering an absolute measure of measurement error at the individual level. For interpretation, SEM values were considered in relation to the

scale's variability (e.g., *SD*), with smaller values reflecting more precise measurement, whereas larger values (>0.5 *SD*) indicate increased uncertainty in individual scores [9].

Construct Validity

Construct validity was assessed using two complementary approaches. Spearman's rank correlations were used to examine the strength and direction of the association between the Pic-ADL (total and domain scores) and established questionnaires measuring similar constructs (A-IADL-Q, B-ADL, SCD-Q, and PDQ). Correlation strength was classified as weak (r_s<.30), moderate (.30–.59), or strong (≥.60) [10]. Agreement was assessed using Kendall's coefficient of concordance (*W*) to assess the extent to which the instruments ranked individuals in a similar order, providing a measure of agreement in relative ordering and interpreted according to Landis and Koch's [11] benchmarks (as implemented for Kendall's *W* in the R package effectsize [12]): .40–.59 = moderate, ≥.60=substantial, and ≥.80 strong to excellent agreement. In addition, associations with informant-based measures (B-ADL and IQCODE) were examined separately. Correlation/concordance with self-report measures was Bonferroni-adjusted to α=.00125 (α=.05/40) and with informant-based measures to α=.0025 (α=.05/20).

Dimensionality

To examine the underlying structure of the Pic-ADL, an exploratory factor analysis (EFA) was conducted on all items simultaneously using principal axis factoring (PAF). This approach was chosen due to its robustness to potential deviations from multivariate normality and its suitability for identifying latent constructs in questionnaire data. Sampling adequacy was assessed using the Kaiser–Meyer–Olkin (KMO) measure and Bartlett's test of sphericity. Factor retention was initially evaluated based on eigenvalues greater than 1 and inspection of the scree plot.

Known-Groups Validity

Known-groups validity (controls vs. mild NCD vs. major NCD) was analyzed using ANCOVA with age and education as covariates and Pic-ADL total/domain scores as dependent variables. Due to violations of normality and homogeneity of variance, bootstrapping (5,000 resamples, BCa intervals) was applied. Differences in the DemTect were assessed using the same method (Bonferroni-adjusted α=.0045 (α=.05/11)). To identify specific group differences, we conducted post-hoc analyses based on Bonferroni-corrected independent sample *t*-tests with bootstrapping.

Criterion validity: Diagnostic performance

Diagnostic accuracy, as an indicator of criterion validity, was evaluated by receiver operating characteristic (ROC) analysis of the Pic-ADL total score. The area under the curve (AUC) indicated discriminative ability (0.5=random, 1.0=perfect prediction accuracy [13]). Optimal cut-offs distinguishing controls/mild NCD from major NCD were determined using the Youden index (J = sensitivity + specificity – 1) and the closest top-left threshold ($d = \sqrt{(1 - sensitivity)^2 + (1 - specificity)^2}$). To further characterize the severity of impairment on the Pic-ADL total score, impairment levels were graded using cut-offs derived from the descriptive statistics of the control group. Furthermore, to more clearly distinguish between subjective cognitive complaints and functional ADL impairment, the total Pic-ADL score was subdivided into two subscores: a functional ADL score and a cognitive score**.** The functional ADL score comprised all items from the 'Technology', 'Personal Hygiene', 'Healthcare', 'Household', 'Mobility', 'Finances', and 'Social Life' domains, whereas the cognitive score included all items from the 'Cognitive Abilities' domain. ROC-based procedures were applied to both subscores to estimate their diagnostic accuracy and to derive corresponding cut-off values. Items from the 'Other' domain were not included in these subscores but are reported descriptively.

Supplementary Text 5. Influence of Item Captions on Psychometric Properties

To examine whether scale captions are necessary for adequate comprehension of the picture-based items, we compared a version of the Pic-ADL with scale captions to a version without captions. This analysis aimed to determine whether item understanding depends on the presence of verbal labels, which is particularly relevant for individuals with language-related limitations. To ensure that participants in the caption and no-caption conditions were comparable with respect to demographic and cognitive characteristics, we used 1:1 nearest-neighbor propensity score matching. Propensity scores were estimated via logistic regression, including age, years of formal education, and DemTect total score as matching parameters. Matching was conducted without replacement, applying a caliper width of 0.3 standard deviations of the logit of the propensity score [14, 15]. To examine group differences between the caption and no-caption conditions, paired *t*-tests were conducted for the Pic-ADL total and domain scores in the matched cohort (α=.05).

To investigate whether item captions influence the scale's psychometric properties, we compared versions with and without captions in a matched subsample of controls. After matching, 14 well-balanced pairs were retained. Balance diagnostics indicated a good matching between the two groups. Across matched pairs, paired *t*-tests detected no statistically significant differences between the caption and no-caption conditions for the Pic-ADL total score or for any of the Pic-ADL domains (all p>.05). Importantly, correlations of the Pic-ADL total score with external validation measures remained significant in both groups (B-ADL self-report, SCD-Q, and PDQ; p<.05), suggesting that the convergent validity of the Pic-ADL is preserved regardless of whether captions are present.

Supplementary Table 1. Cognitive Tests and Respective Cognitive Domain

Cognitive domain	Cognitive test
**Executive function**	
Inhibition	Stroop Interference
Cognitive flexibility and set shifting	CERAD-Plus TMT-B
Phonemic fluency	CERAD-Plus S-words
Working memory	WMS-R Digit Span Backward
**Perceptual-motor function**	
Visuoconstruction	CERAD-Plus Figures Copy
Visual perception	VOSP Cube Analysis
**Complex attention**	
Processing speed	CERAD-Plus TMT-A
Selective attention	Stroop Word & Stroop Color
**Learning and memory**	
Verbal learning	CERAD-Plus Word List Learning
Long-term memory	CERAD-Plus Word List Recall/ Recognition
Short-term memory	WMS-R Digit Span Forward
Visuospatial memory	CERAD-Plus Figures Recall
**Language**	
Object naming	CERAD-Plus Boston Naming Test
Semantic fluency	CERAD-Plus Animals

Abbreviations: CERAD, Consortium to Establish a Registry for Alzheimer's Disease; Stroop, Stroop Color-Word-Interference Test; TMT-A, Trail Making Test A; TMT-B, Trail Making Test B; VOSP, Visual Object and Space Perception Battery; WMS-R, Wechsler Memory Scale – Revised.

Supplementary Table 2. Feasibility, Acceptability, and Internal Consistency of the Pic-ADL in Neurological Patients

	Mean	Median	*SD*	Skewness	Minimum	Maximum	Floor effect (%)	Ceiling effect (%)	Cronbach's α
Technology	1.22	0.88	1.14	0.70	0.00	4.00	13.0	1.5	.93
Personal Hygiene	0.32	0.00	0.58	1.73	0.00	2.00	71.4	0.0	.72
Healthcare	0.49	0.00	0.73	1.73	0.00	3.00	55.1	0.0	.71
Household	0.63	0.42	0.72	0.93	0.00	2.25	38.2	0.0	.75
Mobility	1.02	0.67	0.98	0.77	0.00	4.00	25.7	1.4	.73
Finances	0.42	0.00	0.72	1.93	0.00	3.00	58.0	0.0	.86
Social Life	0.79	0.50	0.94	1.44	0.00	4.00	41.4	1.4	.66
Cognitive Abilities	1.42	1.21	0.91	0.73	0.00	3.76	1.4	0.0	.95
Other Issues	1.49	1.50	1.01	0.30	0.00	4.00	8.6	1.4	.83
Pic-ADL total	1.06	0.85	0.74	0.79	0.10	2.95	0.0	0.0	.97

NOTE: Total and domain scores are calculated relative to the number of items completed, with a maximum score of four points. Therefore, the Pic-ADL total score represents an average score.

Abbreviations: Pic-ADL, Picture-based assessment of subjective deficits in cognition and Activities of Daily Living; *SD*, standard deviation.

Supplementary Table 3. Feasibility, Acceptability, and Internal Consistency of the Pic-ADL in the Control Group

	Mean	Median	*SD*	Skewness	Minimum	Maximum	Floor effect (%)	Ceiling effect (%)	Cronbach's α
Technology	0.32	0.13	0.44	2.45	0.00	2.83	35.1	0.0	.81
Personal Hygiene	0.05	0.00	0.23	5.52	0.00	2.00	94.2	0.0	.84
Healthcare	0.15	0.00	0.41	4.00	0.00	3.50	84.2	0.0	.66
Household	0.09	0.00	0.21	3.01	0.00	1.33	80.5	0.0	.55
Mobility	0.25	0.00	0.42	2.25	0.00	2.33	62.6	0.0	.58
Finances	0.05	0.00	0.13	2.67	0.00	0.60	83.5	0.0	.41
Social Life	0.15	0.00	0.35	2.48	0.00	1.50	80.6	0.0	.56
Cognitive Abilities	0.37	0.24	0.37	1.48	0.00	2.00	18.1	0.0	.90
Other Issues	0.66	0.50	0.64	1.36	0.00	3.25	23.6	0.0	.80
Pic-ADL total	0.29	0.22	0.27	1.37	0.00	1.25	7.4	0.0	.94

NOTE: Total and domain scores are calculated relative to the number of items completed, with a maximum score of four points. Therefore, the Pic-ADL total score represents an average score.

Abbreviations: Pic-ADL, Picture-based assessment of subjective deficits in cognition and Activities of Daily Living; *SD*, standard deviation.

Supplementary Table 4. Results of the Feedback Questionnaire of the Pic-ADL

	**Adjusted Means ± Standard Error [95% CI Bootstrapped]**		**ANCOVA**		
	**Control Group** (*n*=243)	**NCD** (*n*=62)	*F*(df)	*p*-value	$_{p}\eta^2$
Graphics (max. 25 points)	23.20 ± 0.18 [22.83-23.54]	23.28 ± 0.49 [22.14-24.18]	*F*(1, 301)=0.02	.878	.01
Content (max. 10 points)	9.36 ± 0.08 [9.19-9.52]	8.81 ± 0.25 [8.27-9.27]	*F*(1, 301)=5.23	.023	.02
Instructions (max. 15 points)	14.35 ± 0.12 [14.09-14.59]	13.89 ± 0.35 [13.16-14.50]	*F*(1, 301)=1.95	.164	.02
Completion time (max. 5 points)	4.72 ± 0.04 [4.63-4.80]	4.61 ± 0.11 [4.36-4.83]	*F*(1, 299)=0.81	.370	.01

NOTE: Adjusted means from ANCOVA controlling for age and education. All models used bootstrapped confidence intervals (5,000 resamples). *p*-values below a significance level of α=.05 are considered significant. Feedback Questionnaire: higher scores indicate greater satisfaction with the Pic-ADL.

Abbreviations: CI, Confidence interval; NCD, neurocognitive disorder.

Supplementary Table 5. Standard Error of Measurement of the Pic-ADL

	Based on Cronbach's α	
**Pic-ADL**	**½ *SD***	**SEM**
**Technology**	0.38	0.30
Control group	0.22	0.19
Patients	0.57	0.30
**Personal Hygiene**	0.18	0.17
Control group	0.12	0.09
Patients	0.29	0.31*
**Healthcare**	0.26	0.28*
Control group	0.21	0.24*
Patients	0.37	0.39*
**Household**	0.22	0.22
Control group	0.11	0.14*
Patients	0.36	0.36
**Mobility**	0.34	0.37*
Control group	0.21	0.27*
Patients	0.49	0.51*
**Finances**	0.20	0.17
Control group	0.07	0.10*
Patients	0.36	0.27
**Social Life**	0.30	0.33*
Control group	0.18	0.23*
Patients	0.47	0.55*
**Cognitive Abilities**	0.35	0.15
Control group	0.19	0.12
Patients	0.46	0.20
**Other Issues**	0.41	0.32
Control group	0.32	0.29
Patients	0.51	0.42
**Pic-ADL total**	0.27	0.12
Control group	0.14	0.07
Patients	0.37	0.13

NOTE: *SEM > ½ *SD*.

Abbreviations: ICC, intraclass correlation coefficient; Pic-ADL, Picture-based assessment of subjective deficits in cognition and Activities of Daily Living; *SD*, standard deviation; SEM, standard error of measurement.

Supplementary Table 6. Convergence and Agreement between the Pic-ADL and Existing Instruments (Spearman's Rank Correlation Coefficient and Kendall's Coefficient of Concordance) in the Combined Sample

	**A-IADL-Q**		**B-ADL self-report**		**SCD-Q**		**PDQ**	
	r_s	*W*	r_s	*W*	r_s	*W*	r_s	*W*
**Technology**	.37***	1.00***	.52***	.83***	.57***	.74***	.52***	.91***
**Personal Hygiene**	.08	1.00***	.35***	.99***	.30***	.83***	.29***	.94***
**Healthcare**	.15	1.00***	.46***	.96***	.40***	.85***	.42***	.96***
**Household**	.25**	1.00***	.46***	.96***	.48***	.81***	.47***	.95***
**Mobility**	.31***	1.00***	.47***	.92***	.53***	.80***	.46***	.94***
**Finances**	.28***	1.00***	.42***	1.00***	.42***	.83***	.41***	.96***
**Social Life**	.22**	1.00***	.56***	.93***	.54***	.84***	.53***	.96***
**Cognitive Abilities**	.37***	1.00***	.67***	.92***	.77***	.69***	.70***	.90***
**Other Issues**	.34***	1.00***	.52***	.56***	.58***	.59***	.57***	.88***
**Pic-ADL total**	.42***	1.00***	.69***	1.00***	.77***	.58***	.71***	.88***

NOTE: After Bonferroni correction, *p*-values below a significance level of α=.00125 are considered significant. ****p*<.000025, ***p*<.00025, **p*<.00125.

Abbreviations: A-IADL-Q, Amsterdam Instrumental Activities of Daily Living Questionnaire; B-ADL, Bayer Activities of Daily Living; PDQ, Perceived Deficits Questionnaire; Pic-ADL, Picture-based assessment of subjective deficits in cognition and Activities of Daily Living; r_s, Spearman's rank correlation coefficient; SCD-Q, Subjective Cognitive Decline Questionnaire; *W*, Kendall's coefficient of concordance.

Supplementary Table 7. Convergence and Agreement between the Pic-ADL and Existing Instruments (Spearman's Rank Correlation Coefficient and Kendall's Coefficient of Concordance) in the Control Group and Neurological Patients

		A-IADL-Q		B-ADL self-report		SCD-Q		PDQ	
		r_s	*W*	r_s	*W*	r_s	*W*	r_s	*W*
**Technology**	Control group	.23*	1.00***	.39***	.89***	.46***	.69***	.38***	.88***
	Patients	.45**	1.00***	.59***	.63***	.54***	.93***	.56***	1.00***
**Personal Hygiene**	Control group	.10	1.00***	.24**	.98***	.23*	.80***	.13	.93***
	Patients	-.07	1.00***	.34	1.00***	.22	.94***	.23	1.00***
**Healthcare**	Control group	.11	1.00***	.36***	.95***	.28***	.81***	.31***	.94***
	Patients	.12	1.00***	.53***	1.00***	.40*	.99***	.34	1.00***
**Household**	Control group	.15	1.00***	.24**	.98***	.27**	.78***	.21*	.93***
	Patients	.17	1.00***	.57***	.88***	.54***	.94***	.64***	1.00***
**Mobility**	Control group	.21*	1.00***	.27***	.91***	.39***	.76***	.26**	.93***
	Patients	.30	1.00***	.71***	.94***	.65***	.94***	.61***	1.00***
**Finances**	Control group	.19	1.00***	.27***	1.00***	.27***	.79***	.26**	.94***
	Patients	.27	1.00***	.60***	1.00***	.58***	.99***	.56***	1.00***
**Social Life**	Control group	.09	1.00***	.42***	.96***	.40***	.80***	.36***	.94***
	Patients	.15	1.00***	.63***	.83***	.55***	.99***	.55***	1.00***
**Cognitive Abilities**	Control group	.24**	1.00***	.53***	.95***	.64***	.62***	.54***	.87***
	Patients	.35	1.00***	.81***	.83***	.80***	.99***	.79***	1.00***
**Other Issues**	Control group	.21*	1.00***	.43***	.59***	.50***	.50***	.45***	.85***
	Patients	.32	1.00***	.54***	.46***	.56***	.94***	.64***	1.00***
**Pic-ADL total**	Control group	.30***	1.00***	.56***	1.00***	.67***	.49***	.57***	.85***
	Patients	.39*	1.00***	.83***	1.00***	.78***	.94***	.79***	1.00***

NOTE: After Bonferroni correction, *p*-values below a significance level of α=.00125 are considered significant. ****p*<.000025, ***p*<.00025, **p*<.00125.

Abbreviations: A-IADL-Q, Amsterdam Instrumental Activities of Daily Living Questionnaire; B-ADL, Bayer Activities of Daily Living; PDQ, Perceived Deficits Questionnaire; Pic-ADL, Picture-based assessment of subjective deficits in cognition and Activities of Daily Living; r_s, Spearman's rank correlation coefficient; SCD-Q, Subjective Cognitive Decline Questionnaire; *W*, Kendall's coefficient of concordance.

Supplementary Table 8. Correlations of the Pic-ADL with Other Instruments and Demographic Variables

	**Pic-ADL Total Score**	
	r_s	***p*-value**
**DemTect** (max. 18 points)	-.36***	<.001
Control group	-.11	.078
Patients	-.14	.250
**BDI-II** (max. 63 points)	.53***	<.001
Control group	.43***	<.001
Patients	.51***	<.001
**Age** (years)	.46***	<.001
Control group	.24***	<.001
Patients	.11	.389
**Education** (years)	-.28***	<.001
Control group	-.13	.043
Patients	-.20	.101
**Gender** (male/female)	-.04	.493
Control group	-.07	.316
Patients	-.10	.390

NOTE: For gender, a point-biserial correlation was performed. Education represents the total of years of schooling plus vocational and/or tertiary education. After Bonferroni correction, *p*-values below a significance level of α=.0083 are considered significant. ****p*<.00017, ***p*<.0017, **p*<.0083.

Abbreviations: BDI-II, Beck Depression Inventory-II; Pic-ADL, Picture-based assessment of subjective deficits in cognition and Activities of Daily Living; r_s, Spearman's rank correlation coefficient.

Supplementary Table 9. AUC of the Pic-ADL Total, Functional Total, and Cognitive Total Score

	Group comparisons				
**AUC**	Control group vs. mild/major NCD	Control group vs. mild NCD	Control group vs. major NCD	Mild NCD vs. major NCD	Control group/mild NCD vs. major NCD
**Total score**	.87	.79	.92	.81	.91
**Functional total score**	.80	.68	.87	.80	.87
**Cognitive total score**	.88	.80	.92	.82	.92

NOTE: The total score comprises all items; the functional total score includes all ADL items; and the cognitive total score encompasses all items within the domain ‘Cognitive Abilities’.

Abbreviations: AUC, Area Under the Curve; NCD, neurocognitive disorder.

References

1. Wild D, Grove A, Martin M, Eremenco S, McElroy S, Verjee-Lorenz A, et al. Principles of good practice for the translation and cultural adaptation process for patient-reported outcomes (PRO) measures: report of the ISPOR task force for translation and cultural adaptation. Value Health 2005;8(2):94-104. https://doi.org/10.1111/j.1524-4733.2005.04054.x.

2. Smith SC, Lamping DL, Banerjee S, Harwood R, Foley B, Smith P, et al. Measurement of health-related quality of life for people with dementia: development of a new instrument (DEMQOL) and an evaluation of current methodology. Health Technol Assess 2005;9(10):1-iv. https://doi.org/10.3310/hta9100.

3. Martinez-Martin P, Rodriguez-Blazquez C, Abe K, Bhattacharyya KB, Bloem BR, Carod-Artal FJ, et al. International study on the psychometric attributes of the non-motor symptoms scale in Parkinson disease. Neurology 2009;73(19):1584-91. https://doi.org/10.1212/WNL.0b013e3181c0d416.

4. McHorney CA, Tarlov AR. Individual-patient monitoring in clinical practice: are available health status surveys adequate? Qual Life Res 1995;4(4):293-307. https://doi.org/10.1007/BF01593882.

5. Hobart JC, Riazi A, Lamping DL, Fitzpatrick R, Thompson AJ. Improving the evaluation of therapeutic interventions in multiple sclerosis: development of a patient-based measure of outcome. Health Technol Assess 2004;8(9):iii-48. https://doi.org/10.3310/hta8090.

6. Aaronson N, Alonso J, Burnam A, Lohr KN, Patrick DL, Perrin E, et al. Assessing health status and quality-of-life instruments: attributes and review criteria. Qual Life Res 2002;11(3):193-205. https://doi.org/10.1023/a:1015291021312.

7. DeVet HCW, Terwee CB, Mokkink LB, Knol DL. Measurement in medicine: a practical guide. Cambridge: Cambridge University Press; 2011.

8. Terwee CB, Bot SD, de Boer MR, Van der Windt DA, Knol DL, Dekker J, et al. Quality criteria were proposed for measurement properties of health status questionnaires. J Clin Epidemiol 2007;60(1):34-42. https://doi.org/10.1016/j.jclinepi.2006.03.012.

9. Norman GR, Sloan, JA, Wyrwich KW. Interpretation of changes in health-related quality of life: The remarkable universality of half a standard deviation. Med Care 2003;41(5):582-92. https://doi.org/10.1097/01.MLR.0000062554.74615.4C.

10. Fisk JD, Brown MG, Sketris IS, Metz LM, Murray TJ, Stadnyk KJ. A comparison of health utility measures for the evaluation of multiple sclerosis treatments. J Neurol Neurosurg Psychiatry 2005;76(1):58-63. https://doi.org/10.1136/jnnp.2003.017897.

11. Landis JR, Koch GG. The measurement of observer agreement for categorical data. Biometrics 1977;33:159-74. https://doi.org/10.2307/2529310.

12. Ben-Shachar, MS, Lüdecke D, Makowski D. Effectsize: estimation of effect size indices and standardized parameters. J Open Source Softw 2020;5(56):2815. https://doi.org/10.21105/joss.02815.

13. Çorbacıoğlu ŞK, Aksel G. Receiver operating characteristic curve analysis in diagnostic accuracy studies: a guide to interpreting the area under the curve value. Turk J Emerg Med 2023;23(4):195-98. https://doi.org/10.4103/tjem.tjem_182_23.

14. Stuart EA, Rubin DB. Best practices in quasi-experimental designs: matching methods for causal interference. In: Osborne J, editors. Best practices in quantitative methods. Thousand Oaks: SAGE Publications, Inc.; 2008:155-76. https://doi.org/10.4135/9781412995627.

15. Jost ST, Aloui S, Evans J, Ashkan K, Sauerbier A, Rizos A, et al. Neurostimulation for advanced Parkinson disease and quality of life at 5 years: a nonrandomized controlled trial. JAMA Netw Open 2024;7(1):e2352177. https://doi.org/10.1001/jamanetworkopen.2023.52177.
